# Revealing the Effect of Pendant Identity on the Electrochemistry of Non‐Conjugated Redox Active Polymers

**DOI:** 10.1002/cssc.202501121

**Published:** 2025-09-15

**Authors:** Evan Fox, Chen Wang, Mohd Avais, Krista Schoonover, Elizabeth Jergens, David Torres, Khirabdhi Mohanty, Jodie L. Lutkenhaus, Emily B. Pentzer

**Affiliations:** ^1^ Department of Chemistry Texas A&M University College Station TX 77843 USA; ^2^ Artie McFerrin Department of Chemical Engineering Texas A&M University College Station TX 77843 USA; ^3^ Department of Material Science and Engineering Texas A&M University College Station TX 77843 USA

**Keywords:** 2,2,6,6‐tetramethylpiperidin‐1‐oxyl, dopamine, organic batteries, phenothiazine, phthalimide, redox‐active polymers

## Abstract

Understanding charge transfer in redox‐active non‐conjugated polymers is key to unlocking their potential as alternative materials for energy storage. Many factors contribute to charge transfer, such as flexibility of the backbone, redox moiety type, and distance between neighboring redox sites. In a previous work, a series of spatially defined 2,2,6,6‐tetramethylpiperidin‐1‐oxyl (TEMPO)‐containing polymers was reported, with varied spacer lengths between the redox sites. Herein, the synthesis and characterization of spatially defined polymers is reported with the same spacing of three different redox pendants (phenothiazine, phthalimide, and dopamine) and the corresponding electrochemical properties. By doing so, the effect of solution‐polymer interactions (in both the charged and neutral states) is revealed. The apparent diffusion coefficient (*D*_app_), the self‐exchange rate constant (*k*_ex_), and the polymer‐solvent interactions (*χ* and A_2_) of the phenothiazine, phthalimide, and TEMPO polymers are compared. The dopamine‐based polymer exhibits limited solubility, preventing further characterization. *D*_app_ and *k*_ex_ correlate with *χ*, suggesting that solvent favorability enhances charge transfer in the solution state. These findings highlight the important role that polymer‐solvent interactions play in the transfer of electrons, suggesting that a swollen polymer chain conformation promotes solution‐state electron transfer and that solvent favorability promotes charge transfer.

## Graphical abstract

By examining three different redox‐active polymers with identical backbones, this work demonstrates that redox kinetics correlate strongly with polymer‐solvent favorability, in which favorable polymer‐solvent interactions increase the reaction kinetics

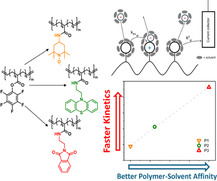

## 1 Introduction

Redox active polymers (RAPs) are emerging as a promising class of materials for use in electrochemical energy storage due to their synthetic tunability,^[^ ^1^, ^2^, ^3^, ^4^, ^5^, ^6^ ^]^ general scalability,^[^ ^7^ ^]^ favorable mechanical properties (e.g., flexibility^[^ ^8^, ^9^ ^]^), and ready access to abundant starting materials^[^ ^10^ ^]^ (e.g., no reliance on precious or geopolitically sensitive feedstocks).^[^ ^11^, ^12^, ^13^ ^]^ RAPs can be divided into two classes: those with either conjugated or non‐conjugated backbones. In conjugated polymers, the backbone serves as the redox‐active component, with characteristic high conductivity along coplanar and overlapping pi orbitals within the backbone.^[^ ^3^, ^14^, ^15^, ^16^ ^]^ Common conjugated RAPs include polyaniline (PANI),^[^ ^17^ ^]^ polypyrrole (PPy),^[^ ^18^ ^]^ and polythiophene (PT).^[^ ^14^, ^19^ ^]^ Initially plagued by low capacity, self‐discharge, and poor cycling stability, performance can be improved by doping as observed for poly(3,4‐ethylenedioxythiophenene)‐poly(styrenesulfonate acid) (PEDOT:PSS)^[^ ^20^, ^21^ ^]^ or via inclusion of redox active side chains.^[^ ^4^, ^22^, ^23^ ^]^

In contrast, non‐conjugated RAPs are composed of an aliphatic polymer backbone with pendant redox‐active groups, such as the nitroxide radical 2,2,6,6‐tetramethylpiperidin‐1‐oxyl (TEMPO), and these have found use in organic batteries, redox flow batteries (RFBs), and other electrochemical systems.^[^ ^24^, ^25^, ^26^, ^27^, ^28^, ^29^, ^30^, ^31^, ^32^, ^33^ ^]^ The TEMPO units undergo reversible oxidation to an oxoamonium cation and reduction back to a nitroxide with charge transfer along the polymer chain facilitated by a hopping mechanism.^[^ ^34^, ^35^, ^36^ ^]^ TEMPO‐containing polymers such as poly(2,2,6,6‐tetramethylpiperidinyloxy‐4‐yl methacrylate) (PTMA) and poly(2,2,6,6‐tetramethylpiperidinyloxy‐4‐yl acrylamide) (PTAm) have garnered significant interest in recent years for their high electron transfer kinetics, stability in air and moisture, and versatility of synthetic preparation. PTAm has demonstrated good electrochemical stability, rapid charge‐discharge performance, and is compatible with aqueous electrolytes.^[^ ^28^, ^37^ ^]^ We previously explored the influence of the polymer backbone on the performance and kinetics of three different, solid‐state TEMPO‐containing polymers in aqueous electrolytes,^[^ ^38^ ^]^ in which favorable water‐polymer interactions led to improved performance. Other studies have shown that the molecular architecture of the polymer backbone and density of TEMPO units along the backbone significantly influence electrochemical performance, with certain structures demonstrating high redox potentials (≈3.6 V vs. Li/Li^+^) and reversible charge storage capacity (>100 mA h/g for 1000 + cycles), good stability, and diffusion‐controlled redox kinetics.^[^ ^39^, ^40^, ^41^ ^]^ Additionally, we recently reported^[^ ^42^ ^]^ the grafting of PTMA onto silica particles resulted in an increase in charge transfer properties near the overlap concentration of a corresponding PTMA solution by promoting interchain electron transfer.

**Figure 1:**
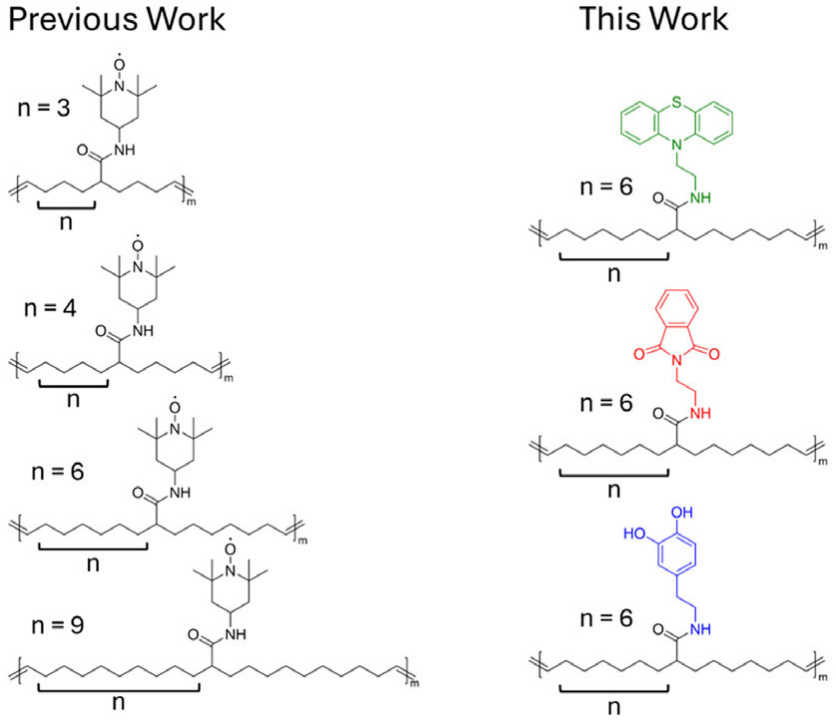
Chemical structures of previously reported (see ref. 29) non‐conjugated RAPS in which the backbone spacing of the TEMPO units was varied (left) and chemical structures of the spatially‐defined redox‐active polymers explored herein, in which spacing is fixed (right).

Our groups recently reported^[^ ^31^ ^]^ a series of non‐conjugated RAPs composed of a hydrocarbon backbone and spatially defined pendant redox active TEMPO units, highlighting the impact of spacing on physical and electrochemical properties (**Figure** **1**). The polymers were synthesized via acyclic diene metathesis (ADMET) polymerization and appended with TEMPO units every 9, 11, 15, and 21 carbons along the backbone via post‐polymerization modification. The self‐exchange rate constant (*k*_ex_) and apparent diffusion coefficient (*D*_app_) of the polymers in the solid‐state were determined using cyclic voltammetry (CV) to reveal an inverse linear relationship between glass transition temperature (*T*_g_) and both log(*D*_app_) and log(*k*_ex_). Ultimately, the polymer with TEMPO groups every 15 carbon atoms had the lowest *T*_g_ and the highest values of *D*_app_ and *k*_ex,app_. Using electron paramagnetic resonance spectroscopy, we observed that there were no spin‐spin interactions between TEMPO units with spacings of 11, 15, and 21 carbons. This can be interpreted as the increased spacing causing a reduction in the likelihood of intrachain electron hopping among nearest‐neighbor pendants in the solid‐state.

To fully understand and leverage the potential of non‐conjugated RAPs for electrochemical energy storage, the impact of the redox‐active pendant group must be defined. Beyond TEMPO, phthalimide, phenothiazine, and dopamine redox units are commonly used. Phthalimides are precursors in the synthesis of several fine chemicals and can undergo a reversible one‐electron redox process of one of the imide carbonyls to a radical anion. Phthalimides have been incorporated onto polyvinylbenzyl,^[^ ^43^ ^]^ polyethyleneoxide,^[^ ^44^ ^]^ and polymethacrylate^[^ ^45^ ^]^ backbones, demonstrating a reversible redox potential of −1.77 V^[^ ^46^ ^]^ to −1.89 V^[^ ^43^ ^]^ versus AgNO_3_/Ag. Recently, Alessandri et al.^[^ ^47^ ^]^ computationally predicted the effects of different backbones on the apparent diffusion coefficient (*D*_app_) of phthalimide‐containing polymers in the solid state using computational modeling, suggesting that the poly(2,3‐epoxy‐propyl) backbone would show the highest value of *D*_app_ due to a good balance of the inner reorganization energy and electronic coupling. Phenothiazines are used in pharmacology applications, and can undergo a reversible single‐electron redox process to produce a radical cation (about 0.3 V vs Fc^+^/Fc), as well as a second single‐electron process to a di‐cation that is generally irreversible (about 1 V vs Fc^+^/Fc).^[^ ^48^ ^]^ Functionalization of the phenothiazine rings with methoxy groups can be used to stabilize the dication.^[^ ^6^ ^]^ Phenothiazine‐containing polymers have demonstrated ultra‐high cycling stability, which has been attributed to the ability of the phenothiazine group to π bond and dimerize, stabilizing the radical cation of the first oxidized state.^[^ ^49^ ^]^ It was later shown that this dimerization process could be disrupted with bulky groups, conformational flexibility, spacing between pendants, and tailoring solubility in the electrolyte.^[^ ^50^, ^51^, ^52^, ^53^ ^]^ Notably, one study examined the effect of spacer length between the phenothiazine pendant group and polymer backbone, showing that increased length reduces electron self‐exchange but improves cycling stability and self‐discharge.^[^ ^54^ ^]^ Bio‐derived redox units, such as catechol and its derivatives (e.g., dopamine), undergo a reversible two‐electron redox process, typically through a proton‐coupled mechanism (e.g., proton‐coupled electron transfer). Polyvinylcatechols have demonstrated redox potential of around 0.4 V (vs Ag/AgCl), a high capacity (>300 mAh g^−1^), and an energy density (1200 Wh kg^−1^),^[^ ^9^, ^55^ ^]^ highlighting the usefulness of the bioderived dopamine units. Catechol‐containing polymers have been plagued by solubility issues^[^ ^56^ ^]^ and difficulty in preparation due to the tendency to cross‐link when oxidized. Whereas prior work has focused upon the effect of varying the backbone identity of a RAP^[^ ^38^, ^47^, ^54^, ^57^, ^58^, ^59^ ^]^ or the spacing of the redox groups, the effect of redox species identity (on the same type of polymer backbone) is less known. By systematically exploring different redox groups attached to a spatially defined architecture, the effect of isolated redox groups and interchain electron hopping (or at least not nearest‐neighbor hopping) can be interrogated to address this knowledge gap.

Herein, we report the synthesis and characterization of three non‐conjugated RAPs with different redox‐active pendant groups along the hydrocarbon backbone (every 15 carbon atoms). To do so, we leverage our previously reported ADMET polymerization approach,^[^ ^31^ ^]^ along with an activated ester. These polymers allowed for the investigation of how different redox pendants influence the physical and chemical properties of the same polymer backbone, specifically through their varied polymer‐solvent interactions. The composition of the polymers was confirmed using ^1^H and ^13^C nuclear magnetic resonance (NMR) and Fourier transform infrared (FTIR) spectroscopies, and the thermal properties were determined by differential scanning calorimetry (DSC) and thermogravimetric analysis (TGA). Electroanalytical testing of the polymers was performed in solution, and the charge transfer kinetics and diffusion coefficients are reported. The Flory–Huggins interaction parameter of the analyzed polymers was calculated to understand how the polymer chain behaves in solution during electrochemical testing in the oxidized and reduced states. Diffusion coefficients are discussed in relation to polymer solvent interaction parameters. We observed a trend between charge transfer (*D*_app_, *k*_ex_) and polymer‐solvent interaction parameters (*χ* and A_2_), in which it was shown that the polymer with the most favorable solvent interactions had the best electron transfer kinetics. This observed trend was opposite of expectations based on the Einstein–Stokes equation, as the increased hydrodynamic radius associated with increased solvent favorability would be expected to hinder diffusion. Instead, our results suggest that polymer chains existing in a more swollen conformation exhibit an increase in electron transfer kinetics.

## 2 Results and Discussion

**Figure 2:**
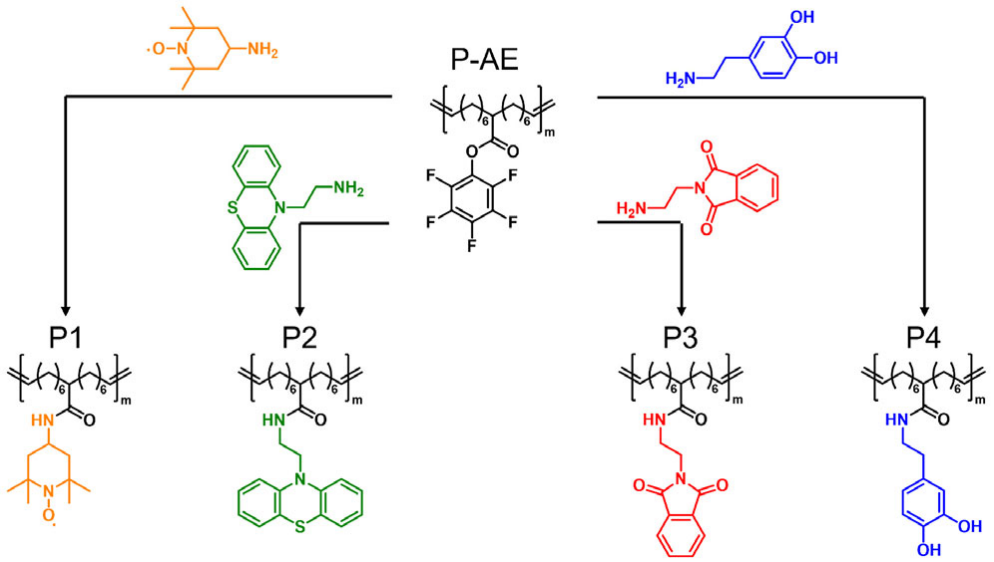
Scheme for preparation of spatially defined non‐conjugated RAPs with pendant redox active units every fifteen carbon atoms. P1 = TEMPO‐containing polymer; P2 = phenothiazine‐containing polymer; P3 = phthalimide‐containing polymer; and P4 = dopamine‐containing polymer.

Polymers with redox‐active units every 15 carbons along the hydrocarbon polymer backbone were prepared by post‐polymerization modification of a parent polymer with pendant activated ester units prepared by ADMET (***P*** **‐AE**), as shown in **Figure** **2**. The redox active units studied are phenothiazine, phthalimide, and dopamine, chosen for their prior use in non‐conjugated RAPs, ready synthetic access to analogs with primary amines, and complementary redox reactions (*n*‐type and *p*‐type). Phenothiazine is a *p*‐type unit and upon oxidation the radical cation formed is compensated by anions; in contrast, phthalimide and dopamine are *n*‐type units, forming anions upon reduction that are compensated by cations. For reference, these polymers were compared to the previously reported^[^ ^31^ ^]^ TEMPO‐containing polymer (**P1**), also a *p*‐type pendant (i.e., producing a cation upon oxidation, which is compensated by an anion).

**Figure 3:**
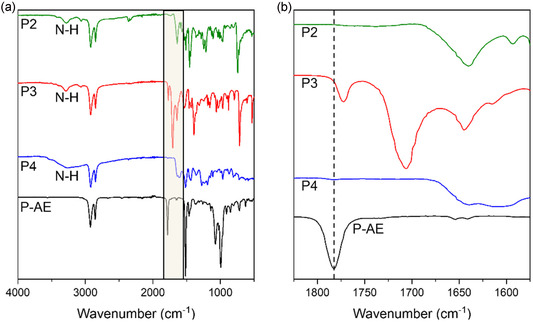
a) FTIR spectra and b) zoom‐in of the carbonyl region for ***P*** **‐AE** (black), phenothiazine‐containing polymer (**P2**, green), phthalimide‐containing polymer (**P3**, red), and dopamine‐containing polymer (**P4**, blue).

Derivatives of the redox active units bearing primary amines were used to modify ***P*** **‐AE** in solution using triethylamine as a base, with full conversion of the activated ester occurring within 24 hours. Consumption of the activated ester to the amide was monitored using ^19^F NMR spectroscopy as shown in Figure S1, Supporting Information, with complete consumption established by the disappearance of the pentafluorophenyl ester peaks (and formation of pentafluorophenol). Functionalization of the polymers was further confirmed using FTIR spectroscopy. As seen in **Figure** **3**, the carbonyl stretching frequency of the activated ester of ***P*** **‐AE** is present at 1787 cm^−1^, and the stretching frequencies of the amide carbonyls of **P2**, **P3**, and **P4** are observed from 1640–1645 cm^−1^. Additionally, for the phenothiazine (**P2**) and the phthalimide functionalized polymers (**P3**), a broad signal indicating a secondary amide N—H stretch is observed at 3285 cm^−1^. The same signal is weakly observed in the dopamine functionalized polymer (**P4**), as this region is dominated by the broad O—H stretching frequency. Noticeably, **P3** shows additional carbonyl bands due to the asymmetric and symmetric C=O stretching frequencies of the cyclic imide. Importantly, the characteristic sharp carbonyl peak at ≈1730–1700 cm^−1^ and broad stretch at 3300–2700 cm^−1^ of a carboxylic acid are absent, indicating the hydrolysis product is not formed. The ^1^H NMR spectra of the polymers show broad signals corresponding to the C—H bonds of the pendant groups and polymer backbone (Figure S2–S4, Supporting Information). The synthesis of **P2** and **P3** was further confirmed using ^13^C NMR spectroscopy, with spectra for **P4** unable to be obtained due to **P4**'s poor solubility. All polymers had M_n_ between 8.5–9 kDa with Ð between 1.6–1.9, as determined by size exclusion chromatography against polystyrene standards (Figure S5, Supporting Information).

The identity of the pendant group dictated the color and solubility of the polymers. When dried, **P2** was a hunter green shiny solid, **P3** was an off white/faint yellow rubbery solid, and **P4** was a red–brown sticky solid. **P2** was soluble in *N*,*N*‐dimethyl formamide (DMF), dimethyl sulfoxide (DMSO), tetrahydrofuran (THF), and chloroform and exhibited solvatochromism. **P2** was found to be insoluble in acetonitrile, dimethyl carbonate, and diglyme. **P3** was fully soluble in DMSO and DMF and sparingly soluble in chloroform and THF, consistent with literature reports^[^ ^43^, ^60^, ^61^, ^62^ ^]^ of other phthalimide‐containing polymers. Disappointingly, **P4** was insoluble in most organic solvents and water and only sparingly soluble in DMSO and THF. This is in contrast to dopamine‐containing polymethacrylates, which are soluble in DMF and methanol.^[^ ^56^ ^]^ This difference in the solubility may be attributed to the presence of both the hydrocarbon backbone and dopamine pendant groups of **P4**, which have orthogonal solubility, e.g., with a good solvent for one being a poor solvent for the other. This is supported by reports of the solubilities of copolymers containing varying levels of pendant dopamine groups, with good solubility only observed at higher levels of dopamine.^[^ ^63^ ^]^ Based on the mentioned report, this insolubility could possibly be overcome by using a polymer backbone with fewer hydrocarbon spacers between dopamine groups. For example, our groups previously reported^[^ ^31^ ^]^ backbones with TEMPO pendants spaced every 9 or 11 carbons, which may provide more favorable solubility.

**Figure 4:**
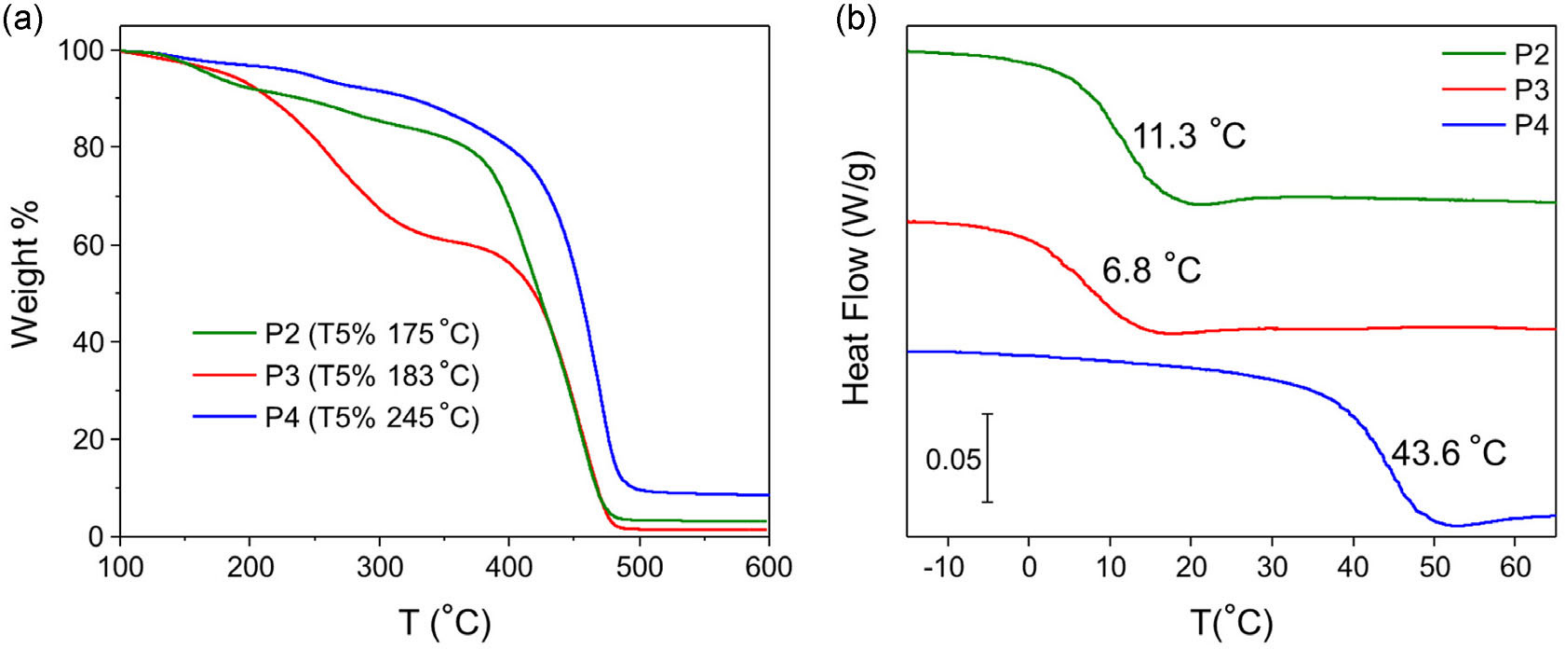
a) TGA weight loss profiles and b) DSC traces of polymers **P2** (green), **P3** (red), and **P4** (blue). DSC thermograms show the second heating cycle, and *T*_g_ values are listed for each polymer.

The thermal properties of the polymers were determined using TGA and DSC. **Figure** **4**a shows the weight loss profiles of all polymers, revealing stability to at least 175 °C, and **P4** losing mass only above 240 °C, which is consistent with literature values for dopamine functionalized polymers.^[^ ^56^ ^]^ Figure 4a shows the DSC traces of the polymers; all had *T*_g_ values above that of **P1** (−13.8 °C), the previously reported^[^ ^31^ ^]^ TEMPO‐containing polymer with the same pendant group spacing. Of the newly prepared polymers, **P4** had the highest *T*_g_ of 43.6 °C, which can be attributed to strong intermolecular interactions, e.g., hydrogen bonding, between dopamine units. However, this value is still lower than that of dopamine‐functionalized methacrylamide (*T*_g_ = 82.5 °C),^[^ ^63^ ^]^ as expected based on the flexible backbone. **P2** and **P3** had *T*_g_ values of 11.3 °C and 6.8 °C, respectively, which are ≈20 °C higher than the TEMPO counterpart; this can be attributed to π–π interactions between aromatic rings of the pendant groups. Notably, the *T*_g_ of **P2** is drastically lower than that reported for phenothiazine functionalized polyvinyl benzene (*T*_g_ ≈ 180 °C^[^ ^64^ ^]^), and the *T*_g_ of **P3** is much lower than that reported for polyvinyl and polymethacrylate derivatives (>120 °C). Thus, the polymers prepared by ADMET all have *T*_g_ values significantly lower than their poly(meth)acrylate and polyvinylbenzene derivatives, supporting that backbone flexibility lowers *T*_g_, regardless of pendant group and their interactions.

**Figure 5:**
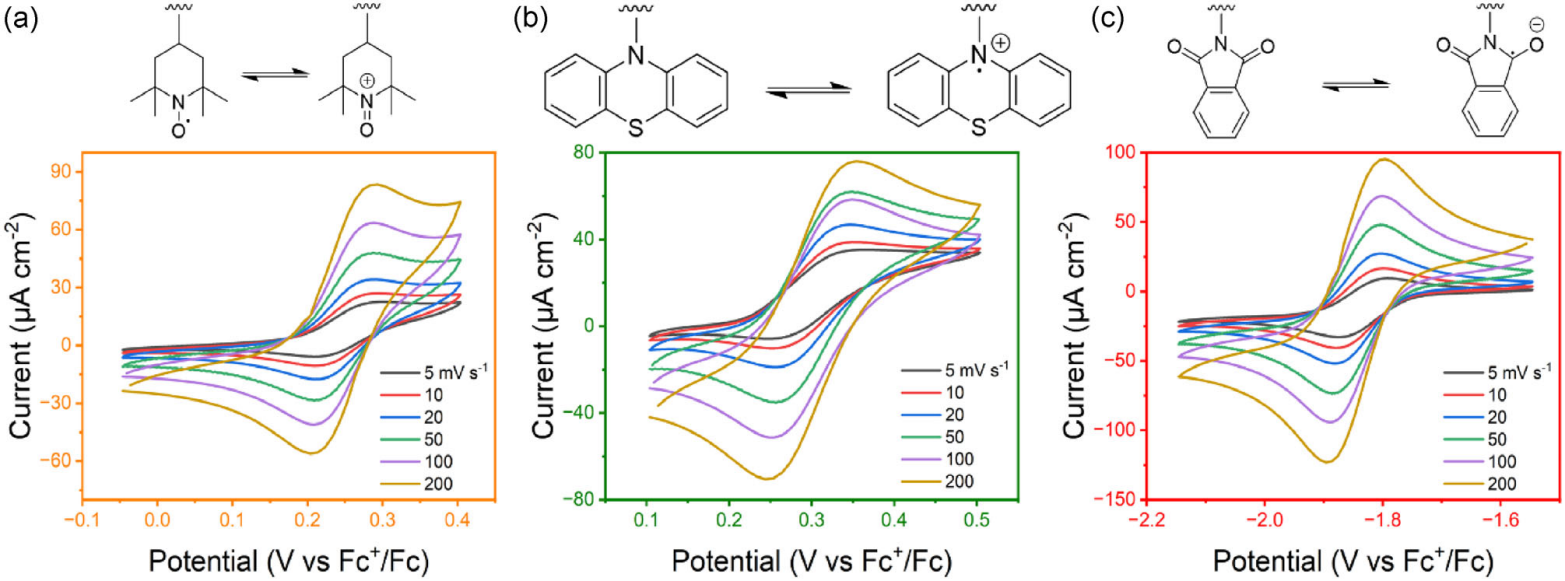
Redox reactions of pendant groups and CV responses of a) **P1** (TEMPO). b) **P2** (phenothiazine), and c) **P3** (phthalimide). CV setup: 2 mM solution (by repeating unit) in 0.5 M TBAPF_6_ in DMF, 3 mm diameter glassy carbon working electrode, platinum wire counter electrode, and silver wire reference electrode. Working potentials are converted using a ferrocene internal reference.

To test how different pendant groups on the same backbone affect the redox properties of the polymers, CV was conducted on 2 mM solutions (by repeating unit concentration) in 0.5 M TBAPF_6_^[^ ^65^, ^66^ ^]^ in DMF at scan rates from 5–200 mV s^−1^. Other solvents such as acetonitrile, dichloromethane, diglyme, and DMSO were tried, but only DMF could dissolve **P1**, **P2**, and **P3**. **P4** was not sufficiently soluble in any of the examined electrolytes and, therefore, its electrochemical properties could not be examined. A different salt, tetrabutylammonium perchlorate (TBAClO_4_), was tested but found to be incompatible with **P3** (i.e., the electrochemical response was irreversible). Glassy carbon was used as the working electrode, platinum wire as the counter electrode, silver wire as the quasi‐reference electrode, and ferrocene as an internal reference (**Figure** **5**). All three polymers show a single pair of peaks, indicating a single‐electron reaction. The half‐wave potential (E_1/2_) of the three polymers (0.249 V for **P1**, 0.306 V for **P2**, −1.83 V for **P3**, all vs Fc^+^/Fc) corresponds with the working potential of their redox centers (TEMPO,^[^ ^67^ ^]^ phenothiazine,^[^ ^57^ ^]^ and phthalimide^[^ ^43^ ^]^). In comparison to a prior study, poly(3‐vinyl‐*N*‐methylphenothiazine) (PVMPT), a polymer with adjacent phenothiazine groups, exhibited peak splitting due to stabilization of the charged phenothiazine group by neighboring neutral ones through π–π interactions.^[^ ^49^ ^]^ Here, we posit that **P2** does not show peak‐splitting because the increased pendant spacing prevented the stabilization effect (i.e., no adjacent phenothiazine groups). This result corresponds with other reports of hindered stabilization effect through the use of bulky substituents.^[^ ^50^ ^]^ **P2** was tested for 100 cycles (Figure S6, Supporting Information) to examine cycling stability, and no degradation or dimerization was observed. Overall, the polymers retain the electrochemical functionality of the appended redox center. The peak separation (Δ*E*_p_) of the three polymers at 10 mV s^−1^ is 80 mV (**P1**), 95 mV (**P2**), and 81 mV (**P3**) (**Table** **1**). Thus, all three polymers show quasireversible reaction kinetics. Higher values of Δ*E*_p_ indicate increasing irreversibility; here, **P2** shows the greatest irreversibility.

**Table 1:** Physical and electrochemical properties of the tested polymers.

	TEMPO (P1)	Phenothiazine (P2)	Phthalimide (P3)
M_n_ a) (kDa) (Đ)	7.5 (1.89)	8.5 (1.55)	9.0 (1.67)
E_1/2_ b) (V vs Fc/Fc^+^)	0.249 ± 0.007	0.306 ± 0.006	‐1.83 ± 0.01
*D*_app,CV_ c) [cm^2^ s^−1^]	8.9 ± 0.1 × 10^−8^	1.0 ± 0.2 × 10^−7^	2.2 ± 0.5 × 10^−7^
*D*_app,CV_ d) [cm^2^ s^−1^]	6.8 ± 0.8 × 10^−8^	1.1 ± 0.2 × 10^−7^	1.9 ± 0.2 × 10^−7^
Δ*E*_p_ e) [mV]	80 ± 7.2	95 ± 6.1	81 ± 1.1
*k* ^0^ c) [cm s^−1^]	1.2 ± 0.01 × 10^−3^	4.7 ± 0.6 × 10^−4^	1.2 ± 0.1 × 10^−3^
*k* ^0^ d) [cm s^−1^]	1.1 ± 0.07 × 10^−3^	5.1 ± 0.4 × 10^−4^	1.1 ± 0.04 × 10^−3^
*D*_app,CA_ c) [cm^2^ s^−1^]	4.8 ± 0.3 × 10^−7^	8.1 ± 2 × 10^−7^	1.2 ± 0.2 × 10^−6^
*D*_app,CA_ d) [cm^2^ s^−1^]	3.0 ± 0.2 × 10^−7^	6.2 ± 0.3 × 10^−7^	7.8 ± 0.9 × 10^−7^
D_phys,DLS_ b) [cm^2^ s^−1^]	2.7 ± 0.1 × 10^−7^	3.1 ± 0.01 × 10^−7^	1.5 ± 0.02 × 10^−7^
*k*_ex_ c) [cm^−1^ s^−1^]	7.1 × 10^11^	1.7 × 10^12^	3.7 × 10^12^
*k*_ex_ d) [cm^−1^ s^−1^]	1.0 × 10^11^	1.1 × 10^12^	2.1 × 10^12^
*χ* neutral stateg)	0.527	0.468	0.396
*χ* ionized stateg)	0.909	0.468	0.343
*A*_2_ [mol mL g^−2^]	−0.003	0.0007	0.009
*R*_H_ [nm]h)	11 ± 1	6.8 ± 0.4	33 ± 2
*D*_phys,EIS_ [cm^2^ s^−1^]	3.77 ± 0.08 × 10^−9^	8 ± 1 × 10^−9^	9.1 ± 0.9 × 10^−9^

a) Measured using refractive index and polystyrene standard.

b) At the lowest scan rate (5 mV s^−1^).

c) Based on the ionization step.

d) Based on the neutralization step.

e) Scan rate 10 mV s^−1^.

f) Measured using DLS.

g) Calculated in DMF and approximated using Fedors method of group contribution.

h) Obtained using SLS and DLS.

A log–log plot of peak current (*i*_p_) versus scan rate (*v*) is shown in Figure S7, Supporting Information, and the slope was examined to assign the *b*‐value. A *b*‐value of 0.5 indicates a semi‐infinite diffusion‐controlled reaction process, which is expected for a solution state measurement.^[^ ^68^, ^69^ ^]^ Also, b‐values less than 0.5 indicate polymer adsorption from the solution onto the electrode surface, which may be due to solubility changes after ionization.^[^ ^70^, ^71^, ^72^, ^73^ ^]^ We found that the b‐values of the ionization current (oxidation scan of **P1** and **P2**, reduction scan of **P3**) were less than 0.5, which we attribute to partial polymer sorption at the electrode. Because the b‐value recovers to ≈0.5 in the reverse (deionization) scan, there is likely a concomitant change in solubility of the polymer. As all b‐values are relatively close to 0.5, a diffusion‐controlled process for all polymers is assigned.

The CV results were then used to estimate (1) the apparent diffusion coefficient (*D*_app,CV_) using the Randles–Sevcik equation^[^ ^74^, ^75^ ^]^ (Equation 1 and 2) the heterogenous rate constant (k^0^) using the Nicholson method^[^ ^76^ ^]^ (Equation 2). *D*_app_ describes the overall charge diffusion in the polymer solution, and *k* ^0^ describes the rate of charge transfer from the polymer to the glassy carbon surface:

(1) $$i_{p}=0.4463nFAC{\left(\frac{nFvD_{\text{app,CV}}}{RT}\right)}^{\frac{1}{2}}$$

(2) $$k^{0}=\Psi \sqrt{\frac{\pi D_{\text{app,CV}}nvF}{RT}}$$

where *n* is the number of electrons transferred, *A* is the area of the electrode, *F* is Faraday's constant, *C* is the redox site concentration, *v* is the scan rate, *R* is the gas constant, and *T* is the temperature. *D*_app_ is determined from the slope of a plot of *i*_p_ and v^1/2^ and reported in Table 1. To better analyze the data, the resulting diffusion and kinetic parameters are classified by ionization step (oxidation reaction of **P1** and **P2**, reduction reaction of **P3**) and neutralization step (reduction reaction of **P1** and **P2**, oxidation step of **P3**). *D*_app,CV_ was ordered as **P3 **> **P2 **> **P1**, indicating that the phthalimide polymer (**P3)** has the fastest apparent diffusion rate. Our calculated *D*_app,CV_ values lie in the range of typical values of polymer solutions (10^−7^ to 10^−9^ cm^2^ s^−1^).^[^ ^26^ ^]^

Using the Nicholson method, k^0^ was estimated from the dimensionless parameter Ψ and v^1/2^ (see Table 1, Equation 2). Ψ is related to Δ*E*_p_, the voltage difference between the oxidation and reduction peaks; this method is acceptable when Δ*E*_p_ is in the range of 63 < Δ*E*_p_ < 212 mV.^[^ ^76^, ^77^ ^]^ A smaller Δ*E*_p_ indicates better reversibility of the reaction; in this work, the Δ*E*_p_ of all three polymer solutions at all scan rates was between 70–120 mV, indicating quasireversible reaction kinetics (Table S1, Supporting Information).^[^ ^78^ ^]^ The *k* ^0^ values for ionization and neutralization steps were similar for the individual polymer solutions, indicating that the charge transfer kinetics do not change significantly with charge state. **P2** showed the lowest *k* ^0^ value, and **P1** and **P3** had similar values. The *k* ^0^ values of all three polymers fell in the range of 10^−3^–10^−5^ cm s^−1^, which is consistent with multiple reports of other redox‐active polymer solutions.^[^ ^26^, ^79^, ^80^ ^]^

To obtain an alternative estimation of *D*_app_,^[^ ^81^ ^]^ chronoamperometry (CA) was performed, and the Cottrell equation (Equation 3) was applied.^[^ ^81^ ^]^ Stepped potential tests (including CA) provide better estimates of diffusion‐kinetic parameters for homogenous reactions (such as self‐exchange), compared to CV whose results are affected by heterogeneous kinetics.

(3) $$i=\frac{nFAC}{\sqrt{\pi t}}\sqrt{D_{\text{app,CA}}}$$

**Figure 6:**
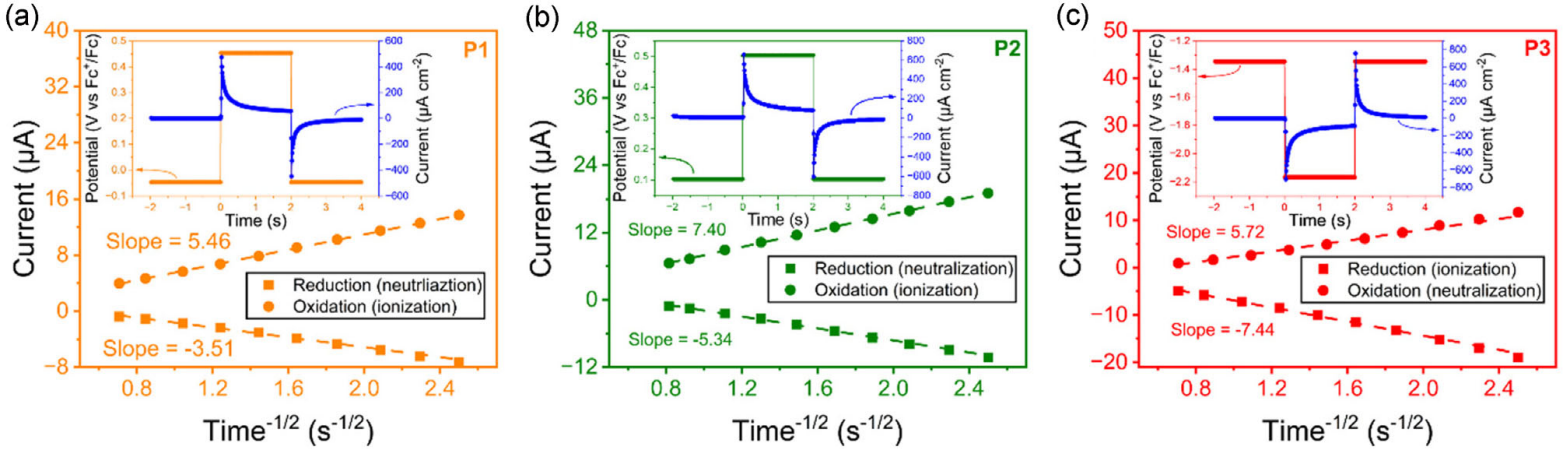
Cottrell plots of a) **P1** (TEMPO), b) **P2** (phenothiazine), and c) **P3** (phthalimide). Dashed lines are linear fits and represent a semiinfinite diffusion process. Insets show the potential steps and current response of each polymer.

The slopes of a Cottrell plot of *i* versus t^−1/2^ are used to determine *D*_app,CA_ (**Figure** **6**, Table 1). The values of *D*_app,CA_ are higher (by about 3–5 times) than corresponding *D*_app,CV_, but follow the same order of **P3 **> **P2 **> **P1**. The *D*_app,CA_ results also lie in the typical reported range of 10^−7^ to 10^−9^ cm^2^ s^−1^.^[^ ^26^ ^]^

In solutions of RAPs, *D*_app_ is the sum of the physical diffusion of the polymer chains (*D*_phys_, physical diffusion coefficient), and the charge transfer within the polymer chain (*D*_et_, electron transfer diffusion coefficient) (Equation 4).^[^ ^82^ ^]^

(4) $$\begin{array}{c} D_{\text{app}}=D_{\text{phys}}+D_{\text{et}} \end{array}$$

To better understand how *D*_phys_ contributes to *D*_app_, dynamic light scattering (DLS) was performed on the polymers in electrolyte solutions (Table 1). DLS measures the fluctuations in scattered light caused by Brownian motion of analytes in the solution to estimate *D*_phys_ using an autocorrelation function. As these polymers are all derived from the same parent backbone, we expect variations in *D*_phys_ to be attributed primarily to differences in the pendant's interactions with the solvent and the pendant's size. *D*_phys,DLS_ of the three solutions were ordered as **P2 **> **P1 **> **P3**, indicating that P2 has the fastest Brownian motion. This order does not follow that of *D*_app_, but the *D*_phys_ of the three polymer solutions lies in the same order of magnitude as *D*_app,CA_. The hydrodynamic radius (*R*_H_) was also obtained from DLS (Table 1) and showed a strong inverse relationship with *D*_phys_, in agreement with the Einstein–Stokes equation. Notably, the trend in *R*_H_ did not follow with *M*_n_, for which it would be expected that **P1** would demonstrate the lowest *R*_H_. The reasoning for this contradiction lies in the varying interactions of the pendant group with the solvent, causing the polymer chain to swell to varying degrees. *D*_phys_ of the ionized polymers could not be measured due to the polymers’ instability in the external measurement environment required by DLS.

To better describe the polymer‐solvent interactions that may influence polymer diffusion, the second virial coefficient (*A*_2_) of each polymer solution was examined and reported in Table 1. *A*_2_ was measured using static light scattering (SLS) of the polymer in electrolyte solution. A positive *A*_2_ value correlates to favorable solvent‐polymer interactions, a negative A_2_ value indicates unfavorable interactions, and the case of *A*_2_ = 0 indicates a theta solvent. **P3** yielded a positive *A*_2_ value (i.e., favorable solvent interactions), **P2**'s *A*_2_ value was near zero, and **P1** yielded a negative *A*_2_ value (i.e., unfavorable solvent behavior). Based on this ranking, it is expected that **P3** would assume a more swollen chain conformation when compared to **P2** and **P1**.

Another indication of polymer‐solvent interactions is the Flory–Huggins interaction parameter, *χ*

(5) $$\chi =\frac{V_{1}}{RT}{\left(\delta_{1}-\delta_{2}\right)}^{2}+0.34.$$

where *V*_1_ is the molar volume of the solvent, *δ*_1_ is the solubility parameter of the solvent, and *δ*_2_ is the solubility parameter of the polymer, as summarized in Table S2–8, Supporting Information. A value of *χ* < 0.5 indicates a favorable solvent‐polymer interaction, *χ* > 0.5 indicates an unfavorable interaction, and *χ* = 0.5 indicates a theta solvent. We previously reported^[^ ^83^ ^]^ the Flory–Huggins solvent interaction parameters of TEMPO‐containing polymers in multiple solvents using both experimental group contribution methods. A separate report^[^ ^38^ ^]^ by us also examined the *χ* of TEMPO‐containing polymers with different backbones used in the solid state with aqueous electrolyte; that study indicated that the polymer with the most favorable solvent interactions with water (lowest *χ*) exhibited accelerated electron transfer kinetics.

Here, *χ* was calculated for **P1, P2,** and **P3** in DMF both in the as‐prepared state (neutral) and after charging (ionized), as summarized in Table 1. In the polymer's neutral state, the *χ* values increased as **P3 < P2 < P1**, with only **P3** and **P2** having *χ* values <0.5. This ranking confirms the observed trend of A_2_ from SLS measurements. In the polymers’ ionized states, the trend of *χ* was the same, but ionized **P1** showed a *χ* value higher than that of its neutral state, indicating that polymer‐solution interactions of **P1** decreased after ionization. The *χ* value of ionized **P3** was lower relative to its respective neutral form, and **P2**'s value remained similar. This change in *χ* can be used to understand solubility changes during oxidation and reduction.^[^ ^83^ ^]^ Specifically, the ionized form of **P1** is expected to be less soluble than its neutral counterpart, whereas the opposite case is expected for **P3**.

With the goal of comparing trends in the electron transfer kinetics and solvent‐polymer interactions, *D*_et_ was calculated using Equation 4 and *D*_app,CA_. With *D*_et_ isolated, the electron self‐exchange rate constant (*k*_ex_), which describes homogeneous charge transfer among pendant groups, was calculated using Dahms–Ruff theory (Equation 6). The results are listed in Table 1.^[^ ^36^, ^84^, ^85^ ^]^

(6) $$k_{\text{ex}}=\frac{6D_{\text{et}}}{C\delta^{2}}$$

**Figure 7:**
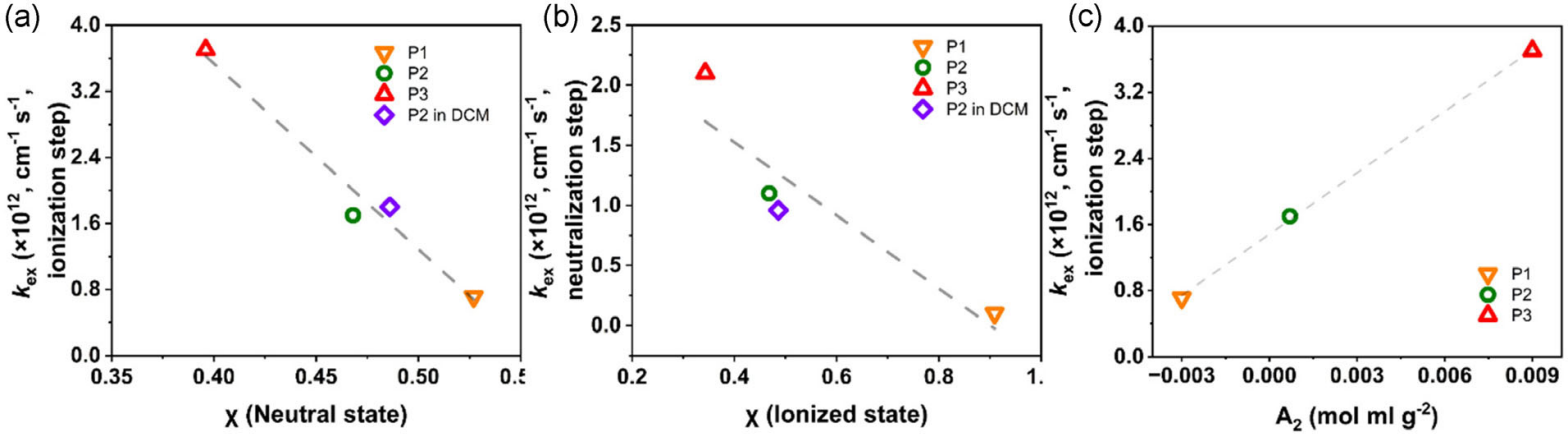
Plots of *k*_ex_ versus polymer‐solvent interactions (*χ* and *A*_2_). a) *k*_ex_ of ionization versus *χ* of neutral state polymer solutions; b) *k*_ex_ of neutralization versus *χ* of ionized polymer solutions; c) *k*_ex_ of ionization versus *A*_2_ of neutral polymer solutions. The orange upside‐down triangle indicates the results of **P1** in DMF, the green circle indicates **P2** in DMF, the red right‐side‐up triangle indicates **P3** in DMF, and the purple diamond indicates **P2** in DCM. The dashed line is a linear fit to guide the eye.

In Equation 6, *δ* is the average redox site distance, estimated as *δ* = (CN_A_)^−1/3^. This estimation does not consider that redox sites are heterogeneously distributed in the polymer solutions (i.e., within a single polymer chain, the pendant‐to‐pendant distance is more representative), but still provides a reasonable estimation of overall redox site distribution in the solution.^[^ ^26^ ^]^ We first consider *k*_ex_ for the ionization step (oxidation for **P1** and **P2**, reduction for **P3**); we compared *k*_ex_ against *χ* in each polymer's neutral state (**Figure** **7**a), which best represents the polymer's initial condition. For the neutralization step (the reverse case), we compared *k*_ex_ against *χ* in each polymer's ionized state (Figure 7b). Because we only have A_2_ of neutral polymer solutions, we compared that with *k*_ex_ for ionization (Figure 7c). The results all show a strong correlation between *k*_ex_ and the solubility characteristics, with better polymer‐solution affinity (lower *χ* or higher *A*_2_) leading to faster kinetics (higher *k*_ex_) for the three polymers reported herein.

For an alternative consideration, we also included the counterion in the polymers’ ionized state when calculating *χ* (Table S9, Supporting Information), but no discernible trend was revealed with *k*_ex_ (Figure S8, Supporting Information). This discrepancy could be attributed to the ionized polymer more likely existing in a solvent‐separated state rather than a contact ion pair state. Solvent mediates the electrostatic interaction between the charged species,^[^ ^86^, ^87^, ^88^, ^89^ ^]^ such that the molecules in closest proximity to the charge group include the solvent. Therefore, the interaction of the ionized polymer and the solvent (Figure 7b) may prove a more consistent interpretation.

To understand if the trends in *k*_ex_ and *χ* held true for another electrolyte, **P2** was tested in dichloromethane (DCM) instead of DMF (Figure S9, Supporting Information and purple diamonds in Figure 7a–b). The result shows that the relationship is valid for both DCM and DMF electrolyte, in which the linear trend is retained. We acknowledge that more polymers and solvents should be examined in the future to fully validate if the relationship is linear or otherwise.

The trends observed in Figure 7 show that polymer‐solvent affinity is a key factor in the homogeneous charge transfer kinetics of RAPs in solution. The charge transfer process in RAPs proceeds by charge hopping from one pendant redox center to another, as related to activation rate constant, *k*_act_, which contributes to *k*_ex_:^[^ ^36^ ^]^

(7) $$k_{\text{act}}=K_{a}\left[{\left(\frac{\pi }{\lambda k_{b}T}\right)}^{\frac{1}{2}}\left(\frac{H_{\text{AB}}^{2}}{\hbar }\right)\exp\left(-\frac{\Delta G^{*}}{k_{b}T}\right)\right]$$

where *K*_a_ is the association constant, *λ* is the reorganization energy, *k*_*b*_ is the Boltzmann constant, *H*_AB_ is the electronic coupling, *ħ* is the reduced Planck's constant, and ΔG* is the activation energy for the transition state. Here, we propose that increasing the polymer‐solvent favorability lowers *λ* and ΔG*, thereby increasing *k*_act_ and *k*_ex_. Specifically, the self‐exchange reaction requires the reorganization of the solvent shell around the pendant upon its ionization and insertion of a compensating ion, for which a more favorable environment will lower the energy barrier for this process.

We also consider the translational diffusion of the polymer chain, as well as the localized motion of pendant groups. *D*_phys_, as measured by DLS, represents the translational diffusion of the polymer chain in solution, but not that of the localized motion of pendant groups. We did not observe an obvious trend with *D*_phys_ and *k*_ex_ or *k* ^0^, suggesting that translational diffusion is less influential. If we, instead, consider the localized motion of pendant groups, we must consider segmental chain mobility, which is influenced by polymer‐solvent interactions and chain conformation. As polymer‐solvent interactions become more favorable, chain relaxation should be enhanced, thus accelerating the charge transfer process. In our previous report for solid‐state non‐conjugated RAPS, we observed a positive correlation between the charge transfer kinetics and the polymer‐solvent interaction parameter.^[^ ^38^ ^]^ We speculate that a similar concept can be applied herein to explain the trend in redox kinetics for our polymer solutions.

**Figure 8:**
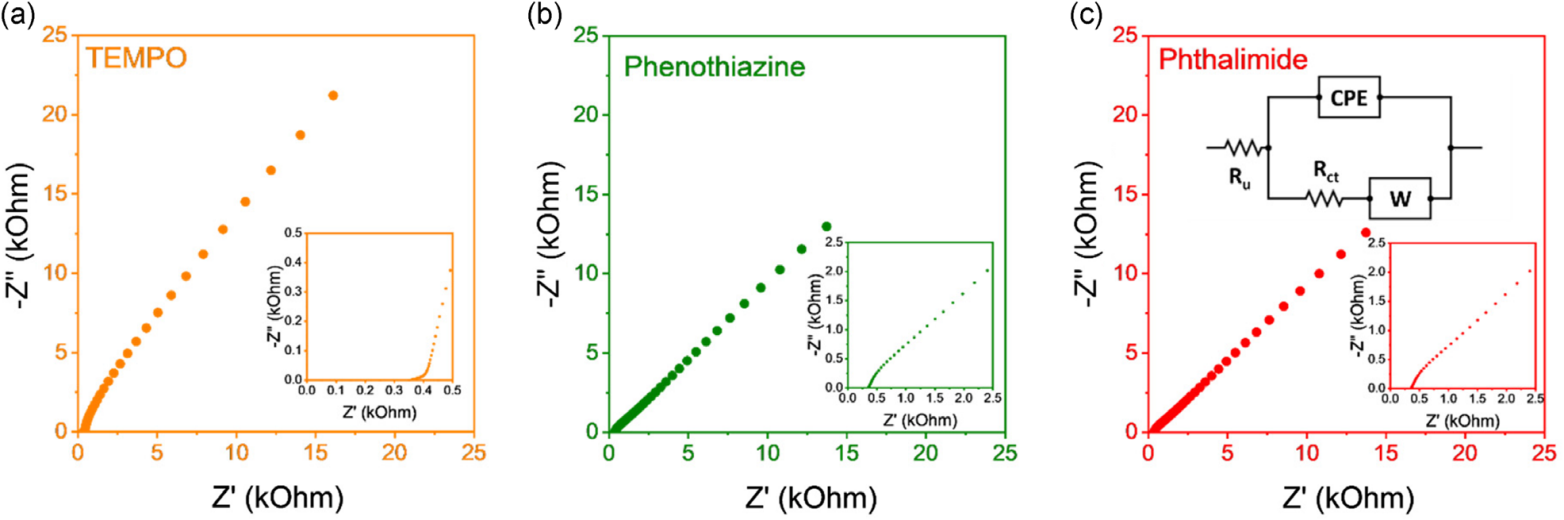
EIS Nyquist plots of a) **P1** (TEMPO), b) **P2** (phenothiazine), and c) **P3** (phthalimide). EIS tests were performed at E_1/2_ for each polymer using a frequency range of 0.1 Hz to 100 kHz and an amplitude of 5 mV. Insets show the high‐frequency regions of the Nyquist plots. Schematic inset in (c) shows the equivalent circuit used to model the EIS results. *R*_u_ indicates the solution resistance, *R*_ct_ indicates the charge transfer resistance, constant phase element (CPE) is a constant phase element that describes the capacitive behavior, and *W* is a Warburg element that captures diffusion‐limited processes.

Impedance testing was also conducted to electrochemically observe the value *D*_phys_ of the polymer near the electrode surface. Electrochemical impedance spectroscopy (EIS) was conducted at the E_1/2_ voltage of each polymer to examine the frequency‐dependent impedance response (**Figure** **8**). All three polymers showed a similar response, with a high‐frequency semicircular segment, followed by a low‐frequency diffusion tail. The equivalent circuit in Figure 8c shows that the semicircular feature is ascribed to the charge transfer resistance (*R*_ct_) and the linear tail segment is associated with the Warburg impedance (*Z*_w_), which describes mass transport limitations of the redox species to the electrode surface considering a semiinfinite diffusion. From equivalent circuit modeling, the Warburg coefficient (*σ*) was determined, allowing for an estimate of the physical diffusion of each redox‐active polymer using Equation 8.^[^ ^90^ ^]^

(8) $$\sigma =\frac{2RT}{n^{2}F^{2}AC\sqrt{2D_{\text{phys,EIS}}}}$$

From Equation 8, the derived diffusion coefficient is noted as *D*_phys,EIS_, and listed in Table 1. *D*_phys,EIS_ describes the diffusion of reacting redox species in the mass transport layer near the electrode surface.^[^ ^90^, ^91^ ^]^ *D*_phys,EIS_ was two orders of magnitude smaller than *D*_app_ or *D*_phys,DLS_, but follows the trend of D_et_ of **P3 **> **P2 **> **P1**. The different values of *D*_phys,EIS_ and *D*_phys,DLS_ are due to how they are measured. Specifically, DLS measures *D*_phys_ via Brownian motion of polymer chains in the bulk solution,^[^ ^92^ ^]^ whereas EIS measures *D*_phys_ via semiinfinite diffusion of polymer chains driven by concentration gradients at the proximity of the electrode surface.^[^ ^93^ ^]^ Overall, a positive correlation between *D*_phys,EIS_ and *χ* is observed (Figure S10, Supporting Information), further supporting the phenomena that higher polymer‐solvent affinity allows for faster kinetics in redox reactions at the electrode surface.

## 3 Conclusion

A series of spatially defined polymers with three different redox active pendant groups were synthesized via amidation of an activated ester polymer prepared by ADMET. The hydrocarbon polymer backbones were appended with pendant phenothiazine, phthalimide, and dopamine units, comparing to the previously reported analog with pendant TEMPO groups. This approach demonstrated the versatility and modularity of our previously published^[^ ^31^ ^]^ in producing spatially defined activated redox groups required to evaluate structure‐property relationships. In supporting electrolyte solutions, the redox‐active polymers displayed quasireversible redox activity and stability over multiple cycles; notably, the dopamine containing polymer was not soluble and therefore its electrochemistry in solution could not be evaluated. The apparent diffusion coefficients and exchange rate constants of the other polymers correlated with solvent favorability (as described by *χ* and A_2_), suggesting that solvent favorability enhances charge transfer in the solution state. These findings challenge the idea that increasing chain size would decelerate reaction kinetics simply due to decreased physical diffusion. Instead, these findings highlight the positive correlation between polymer‐solvent affinity (*χ*) and electron exchange kinetics, in which favorable interactions are recommended because they lead to faster charge transfer.

Future work should focus on the development of non‐conjugated redox‐active polymers with high concentrations of redox‐active units and strongly favorable solvent interactions, without drastic increases in viscosity. Increasing the concentration of redox‐active units (by lowering the molecular weight of the repeat unit or by simply increasing the molarity of the solution) would increase the theoretical capacity and energy density. Meanwhile, as shown herein, favorable solvent interactions are expected to increase the charge transfer kinetics. Notably, increasing polymer concentration commonly increases solution viscosity, especially above the overlap concentration, preventing practical application in, e.g., RFBs. Therefore, enhancing polymer‐solvent interactions may play an important role in not only improving the polymer's solubility but also its charge transfer kinetics.

## Conflict of Interest

The authors declare no conflict of interest.

## Author Contributions

**Emily B. Pentzer** and **Evan Fox** conceived the study. **Evan Fox**, **Chen Wang**, **Mohd Avais**, **Emily B. Pentzer**, and **Jodie L. Lutkenhaus** discussed the results and wrote the manuscript. **Evan Fox** synthesized all polymers and performed characterization. **Evan Fox** calculated Hildebrand solubility parameters and Flory‐Huggins solvent interaction parameters. **Chen Wang** and **Mohd Avais** performed electrochemical testing. Krista Schoonover performed SLS and DLS. **Elizabeth Jergens** synthesized the phenothiazine pendant. **David Torres** synthesized the phthalimide pendant. **Evan Fox** and **David Torres** synthesized the monomer. **Khirabdhi Mohanty** conducted cycling stability test of P2.

## Supporting information

Supplementary Material

## Data Availability

The data that support the findings of this study are available from the corresponding author upon reasonable request.
