# Supplementary material for: Revealing the Effect of Pendant Identity on the Electrochemistry of Non‐Conjugated Redox Active Polymers
Source: ChemSusChem. 2025 Sep 15;18(20):e202501121. doi: 10.1002/cssc.202501121 (PMC12548945; doi:10.1002/cssc.202501121)
Supplement: Supplementary file 1 — Supplementary Material [file CSSC-18-e202501121-s001.pdf]

Supporting Information for

Revealing the Effect of Pendant Identity on the Electrochemistry of Non-conjugated Redox Active Polymers

Evan Fox[a], Chen Wang[b], Mohd Avais[c], Krista Schoonover[a], Elizabeth Jergens[c], David Torres[b], Khirabdhi Mohanty,[b] Jodie L. Lutkenhaus*[bc], Emily B. Pentzer*[ac]

[*] Corresponding Author
[a] Department of Chemistry, Texas A&M University, College Station, TX 77843
[b] Artie McFerrin Department of Chemical Engineering, Texas A&M University, College Station, TX 77843
[c] Department of Material Science and Engineering, Texas A&M University, College Station, TX 77843

*jodie.lutkenhaus@tamu.edu, emilypentzer@tamu.edu

Experimental

Materials – All chemicals were used as received and purchased from Sigma-Aldrich unless otherwise stated. Ditertbutyldicarbonate was purchased from Oakwood Chemical. 2-chloroehtylamine HCl was purchased from TCI. Dopamine HCl was generously donated by the Wooley Group at Texas A&M University.

Instrumentation – FTIR spectroscopy was performed using a JASCO FTIR spectrometer, model FTIR-4600LE MidIR. ^{1}H, ^{13}C, and ^{19}F NMR spectra were recorded on a Bruker 400 spectrometer. Glass transition temperatures were measured by differential scanning calorimetry on a Q200 DSC (TA Instruments) with a heat−cool−heat cycle at a ramp rate of 10 °C/min. The glass transition temperature was taken as the midpoint point of the second heating cycle. Thermogravimetric analysis was performed on a Discovery TGA 550 (TA Instruments), runs were ramped to 100 °C and held for 10 min, and then ramped to 600 °C at rate of 10 °C/min. Molecular weight was obtained using gel permeation chromatography (TOSOH high-temperature GPC with tetrahydrofuran, sample column TSKgel GMH_{HR}-M) using refractive index and polystyrene standard. Dynamic light scattering was performed using a Malvern Zetasizer Nano ZS at 25 ºC, averaging three sets of 10 s scans collected at 173º backscatter. Static light scattering was performed on a ALV/CGS-3 Static Light Scattering goniometer at 20 ºC, collecting data at 10s runtimes in increments of 10º from 30 to 150º. Refractive index increment (dn/dc) was measured using a WYA-3S ABBE Digital Refractometer at 20 ºC, dn/dc taken as the slope of refractive index versus concentration for each polymer solution.

Synthesis and Characterization

Phthalimide pendant synthesis – The phthalimide pendant, 2-(2-aminoethyl) isoindoline-1,3-dione HCl, was synthesized as shown using the two schemes below, over a total of 3 steps. First, 2-chloroethylamine was BOC protected. Second, the BOC protected chloroethylamine was reacted with potassium phthalimide to afford the BOC protected version of the desired pendant, which was deprotected using concentrated HCl.

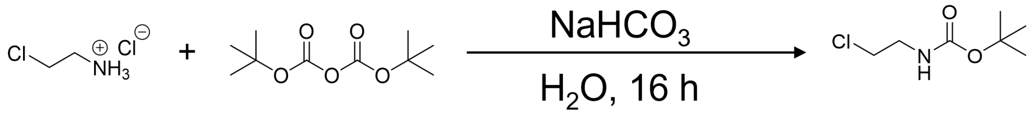

Scheme S1: Reaction scheme for synthesis of BOC protected 2-chloroethylamine.

Tertbutyl (2-chloroethyl) carbamate – The Boc protected 2-chloroethylamine was able prepared on multigram scale using a procedure adapted from literature[1] and is a follows. 2-chloroethylamine HCl (1.1eq) was dissolved in saturated $NaHCO_3$ solution (1.2 mL per mmol of amine) with stir bar. Ditertbutylcarbonate (BOC anhydride) (1eq) was then added to the solution and placed under nitrogen and reacted overnight. The reaction mixture was then extracted with ethyl acetate (3 x 25 mL) and dried over sodium sulfate. The product was then concentrated under vacuum to yield a clear faint yellow oil. Synthesis was able to be scaled to multiple grams with excellent yields. Yield-(91%)

^{1}H NMR (400 MHz, $CDCl_3$): 4.92 (1H, s), 3.58 (2H, t), 3.47 (2H, t), 1.45 (9H, s)

^{13}C NMR (100 MHz, $CDCl_3$): 155.66, 79.78, 44.33, 42.43, 28.34

ESI +: [M+H] 180.075 m/z

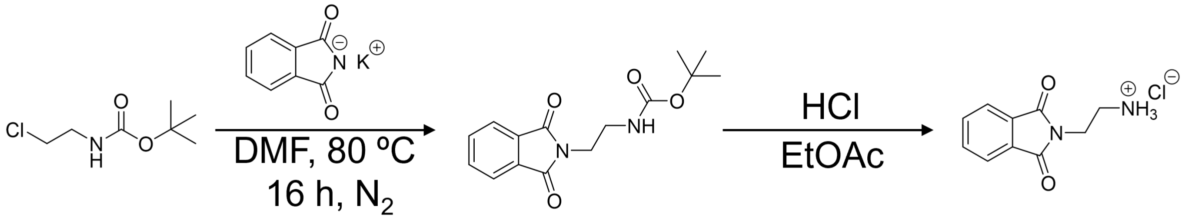

Scheme 2: Synthetic scheme for 2-(2-aminoethyl) isoindoline-1,3-dione HCl.

2-(2-aminoethyl) isoindoline-1,3-dione HCl – tertbutyl (2-chloroethyl) carbamate (10 mmol) was added to a 100 mL round bottom flask with a stir bar and dissolved in DMF. Potassium phthalimide (14 mmol) was then added to the solution. The reaction mixture was refluxed at 80 °C overnight. The reaction mixture was then poured into 100 mL of water and extracted with diethyl ether (3 x 25 mL) and the organic layer washed with brine. Solvent was then removed under vacuum to yield a white solid. Product was verified via ^{1}H NMR and immediately used in the next step without further purification. The white solid was dissolved in minimal ethyl acetate in a round bottom flask with stir bar. ~3 mL of concentrated HCl was then added to the mixture. A white precipitate began to form, and the reaction mixture was left overnight. The next day the reaction

mixture was gravity filtered to collect the solid precipitate and washed with ethyl acetate. The solid was dried under vacuum to afford the pure product as a puffy white solid. Yield – (56%)

^{1}H NMR (400 MHz, DMSO-d6): 8.11 (3H, s), 7.88 (4H, m), 3.85 (2H, t), 3.08 (2H, t)

^{13}C NMR (100 MHz, DMSO-d6): 168.48, 134.83, 132.44, 123.51, 37.82, 35.78

MASS SPEC (+ESI): [M+H] 191.0816 m/z

Phenothiazine Pendant – The phenothiazine pendant 2-(10H-phenothiazin-10-yl)ethan-1-amine was synthesized using previously reported literature procedure.[2]

MASS SPEC (+ESI): [M+H] 243.0947 m/z

Activated Ester Polymer (P-AE) – The activated ester polymer was synthesized via Acyclic Diene Metathesis (ADMET) using procedures we have previously reported[3].

Polymer functionalization – Detailed functionalization procedures can be found below. A general procedure for synthesis of the functional polymer P2 is described.

N6 Phenothiazine Polymer (P2) – The n = 6 activated ester parent polymer (.267 g, 0.637 mmol) was dissolved in ~ 4 mL of dry DCM and added to a 20 mL scintillation vial with a stir bar along with ~3 mL of DMSO to improve solubility and put under nitrogen. The solution was sparged with nitrogen for ~10 min to remove any oxygen. 2-(10H-phenothiazin-10-yl) ethan-1-amine (1.5 eq) was dissolved in ~2 mL of dry DCM and added to the reaction mixture, as well as triethylamine (1.5 eq). The reaction mixture was placed back under nitrogen for 24 h. Conversion from activated ester to the desired amide was tracked using ^{19}F NMR spectroscopy as seen in **Figure S1**. Once the signals associated with the pentafluorophenyl ester no longer appeared, the reaction was considered to be complete. The reaction mixture was placed in a separatory funnel and was washed 5% HCl (2 x 25 mL). The organic layer was then taken and concentrated under vacuum to yield the functionalized polymer as a dark green shiny solid. The polymer was found to be solvatochromatic and varied between dark green in chloroform, purple/pink in DCM, light yellow in DMF, and dark yellow in THF. Yield – (90%)

^{1}H NMR (400 MHz, $CDCl_3$): 7.17 (4H, d) 6.93 (4H, s), 6.12 (1H, s), 5.78 (end group, m) 5.33 (2, m), 4.95 (end group, m), 4.05 (2H, m), 3.60 (2, m), 1.94 (1H, m), 1.91 (4H, m), 1.35 (4H, dm), 1.24 (4H, m), 1.09 (8H, m), 1.04 (4H, m).

^{13}C NMR (100MHz, $CDCl_3$): 177.04, 139.17, 130.32, 129.86, 127.62, 116.15, 48.20, 33.04, 32.59, 29.64, 29.51, 28.89, 27.51, 27.21

N6 Phthalimide Polymer (P3) – The same general procedure described above was followed for the synthesis of P3 with the exception that 2-(2-aminoethyl)isoindoline-1,3-dione HCl was used instead of 2-(10H-phenothiazin-10-yl)ethan-1-amine, and was added directly into the reaction mixture. The polymer was collected as an off white, slightly yellow rubbery solid. The polymer

exhibited solubility in DMSO and DMF, and was mildly soluble in chloroform and THF. It was insoluble in acetone, ethyl acetate, and DCM. Yield – (94%)

^{1}H NMR (400 MHz, $CDCl_3$): 7.76 (4H, m), 6.12 (1H, s), 5.78 (end group, m), 5.31 (2H, s), 4.98 (end group, m), 3.85 (2H, m), 3.56 (2H, m), 1.89 (5H, m), 1.48 (2H, m), 1.24-1.14 (18H, m)

^{13}C NMR (100MHz, $CDCl_3$):168.5, 168.2, 134.2, 131.9, 130.3, 123.3, 37.5, 36.8, 32.9, 32.5, 30.3, 29.6, 29.5, 29.0, 27.6

N6 Dopamine Polymer (P4) – The same general procedure described above was followed for the synthesis of P2 with some variations adapted from literature procedure[4]. Dopamine HCl was used instead of 2-(10H-phenothiazin-10-yl) ethan-1-amine and was added directly into the reaction mixture. Once the amidation was complete the reaction mixture was poured directly into 100 mL 5% HCl. The 5% HCl was decanted off and the solid was collected with THF and precipitated in water. The solid was then collected and dried under vacuum. After drying, the polymer was collected as a red/brown semi translucent crystalline solid. The polymer exhibited insolubility or very poor solubility in organic solvents (DMSO, DCM, DMF, methanol), and was sparingly soluble in THF. Due to poor solubility ^{1}H NMR was only able to be obtained after much difficulty getting P4 into solution amounts needed for testing, with ^{13}C NMR not able to be obtained due to low concentration and poor S/N. Yield – (89%)

^{1}H NMR (400 MHz, DMSO-d_6): 8.68 (2H, s, b), 7.87 (2H, s), 6.63 (1H, d), 6.59 (1H, s), 6.43 (1H, d), 5.80 (end group, m) 5.35 (2H, m) 4.98 (end group, m), 3.21 (2H, m), 2.07 (1H, m), 1.93 (4H, m), 1.50-1.10 (20H, m).

^{13}C NMR (100MHz, $CDCl_3$): Unable to be collected due to poor solubility.

Static Light Scattering – Static light scattering was performed on solutions of each polymer dissolved in HPLC-grade DMF starting at 2 mM concentration and diluting by half 2-4 times for a total of 3-5 concentrations. For the DMF with electrolyte solutions, polymers were dissolved at twice the initial concentration and diluted by added doubly concentration electrolyte solution, which promoted rapid dissolution. Data was analyzed using the Zimm methodology to derive M_w, R_g and A_2.

Dynamic Light Scattering – All DLS measurements were performed over a polymer concentration range of 0.16–5.00 mg/mL with comparable results across that range, indicating all measurements were in the dilution zone. Measurements below 1 mg/mL had decreased signal to noise ratio, so the optimal concentration was selected as 1.25 mg/mL polymer in solution to stay below the predicted overlap concentration and above the noisy regime. A set of samples was also analyzed in electrolyte and was run at similar polymer concentration with an electrolyte concentration of 0.5M $TBAPF_6$ in DMF.

Electrochemistry – The electrochemical tests were carried out in polymer solutions (2 mM by repeat unit) with 0.5M tetrabutylammonium hexafluorophosphate ($TBAPF_6$) in N,N-dimethylformamide (DMF). The tests are done in a three-electrode cell with a 3 mm glassy carbon working electrode, platinum wire counter electrode, and silver rod as the quasi-reference electrode (QRE). All electrochemical tests are done in an argon-filled glove box with oxygen and moisture level below 10 ppm. All working potentials were converted using a ferrocene internal reference. All electrochemistry tests were carried out using a Reference 600 Potentiostat (Gamry Instruments Inc.). The CV tests were carried out first, with scan rates from 5, 10, 20 ,50, 100, 200 mV s^{-1}. The scanning potential window for the three polymers were: for **P1** 0.7 – 1.2 V (vs Ag QRE, oxidation reaction first); for **P2** 0.85 – 1.25 V (vs Ag QRE, oxidation reaction first), for **P3** -0.8 - -1.4 V (vs Ag QRE, reduction reaction first). Then the CA tests were carried out; the potential steps used in CA tests were: for **P1**, 0.7 V for 2s, 1.2 V for 2 s and then 0.7 V for 2 s; for P2, 0.85 V for 2 s, 1.25 V for 2 s, then 0.85 V for 2 s; for P3, -0.85 V for 2 s, -1.4 V for 2 s, then -0.85 V for 2 s (all vs Ag QRE). Lastly the EIS tests were carried out. The EIS was done at $E_{1/2}$ of each polymer (DC voltage = 0.7 V for **P1**, 0.71 V for **P2**, -1.4V for **P3**, all vs Ag QRE), with an AC voltage amplitude of 10 mV and frequency from 0.1 Hz to 100 kHz.

Table S1. The ΔE_p of three polymers at different scan rates.

Scan rate (mV s^{-1})	ΔE_p of **P1** (mV)	ΔE_p of **P2** (mV)	ΔE_p of **P3** (mV)
5	87 ±3.1	114 ± 14	84 ± 0
10	80 ± 7.2	95 ± 6.1	81 ± 1.1
20	74 ± 4.7	89 ±2.1	80 ± 3.1
50	75 ± 4.6	92 ± 1.7	84 ± 3.8
100	82 ± 2.9	98 ± 2.9	92 ± 7.6
200	90 ± 10	110 ± 0	103 ± 15

The Ψ parameter in the Nicholson is derived from ΔE_p from 50-200 mV s^{-1}, using the following equation:[9]

$$\Psi = \frac{-0.6288 + 0.0021(n\Delta Ep)}{1 - 0.0017(n\Delta Ep)}$$

In which, n is the number of electrons transferred.

Calculating Solubility Parameters – Hildebrand solubility parameters (δ) were calculated using the Fedors method with group values for energy of vaporization (ΔE) and molar volume (V_m) obtained from literature[5]. Values used for the nitroxide group and oxoammonium group were those reported by Easley *et al.* in previous work focused on determining solubility parameters of nitroxide radical polymers.[6] Values for the charged state of P3 were estimated for the C-O^-

oxyanion radical by approximating its contributions as a quaternary carbon and an oxygen, similar to relevant literature on approximating contributions of nonstandard groups[6]. P2 has no change in groups or bonds so it has no change using the Fedors method. All solubility parameters were calculated at 298.15 K. Values used for DMF were found from literature.[7]

Calculating Hildebrand solubility parameters using literature values can be somewhat confusing as older literature values of energy contribution are reported in non SI units of cal/mol. This is further compounded that more recent literature uses SI units of kJ/mol and then reports the final solubility parameter (δ) in $MPa^{1/2}$. Calculations were done in cal/mol as the resulting units of δ ($cal^{1/2}\, cm^{-3/2}$) can easily be converted into $MPa^{1/2}$, where 1 $cal^{1/2}\, cm^{-3/2} = 2.0454843\ MPa^{1/2}$.

The calculations for estimating the δ of each polymer was done by breaking down the common backbone for all polymers and respective pendants into the individual molecular groups that compose the structure of their repeat unit. The ΔE and V_m values of the respective groups were totaled into a single value for each pendant and the backbone. For each polymer the values of the respective pendant and the backbone were added to obtain a single value of ΔE and V_m.

For each polymer, the total ΔE and V_m was approximated by adding together the contribution of the backbone and the pendant. The δ was calculated using the formula:

$$\delta = \sqrt{\frac{\Delta E}{V_m}}$$

This value was then converted to $MPa^{1/2}$ to be used to determine χ. For determining χ of the ion pairs the ΔE and V_m for the repeat unit were calculated as if the counter ion was part of the repeat unit itself. The group contribution values for the respective counter ion were added to the total of the repeat unit to obtain the δ and χ of the polymer ion pairs.

Table S2. Fedors group contributions for polymer backbone.

Group	Quantity	E (cal/mol)	E (kJ/mol)	V_m (cm^3/mol)
CH	1	820	3.43	-1
$-CH_2-$	12	1180	4.94	16.1
-CH=	2	1030	4.31	13.5
CONH	1	8000	33.5	9.5
Total		25040	104.8	228.7

Table S3. Fedors group contribution of the TEMPO pendant on P1.

Group	Quantity	E (cal/mol)	E (kJ/mol)	V_m (cm^3/mol)
Nitroxide	1	9680	40.5	-5.3
$-CH_3$	4	1125	4.71	33.5
C	2	350	1.46	-19.2
$-CH_2-$	2	1180	4.94	16.1
CH	1	820	3.43	-1
6 member ring	1	250	1.05	16
Total		18310	76.6	137.5

Table S4. Fedors group contribution of the TEMPO pendant on P1 in the charged state.

Group	Quantity	E (cal/mol)	E (kJ/mol)	V_m (cm^3/mol)
Oxoammonium	1	9680	40.5	-5.3
$-CH_3$	4	1125	4.71	33.5
C	2	350	1.46	-19.2
$-CH_2-$	2	1180	4.94	16.1
CH	1	820	3.43	-1
6 member ring	1	250	1.05	16
Total		9630	54	151.6

Table S5. Fedors group contribution of the phenothiazine pendant on P2.

Group	Quantity	E (cal/mol)	E (kJ/mol)	V_m (cm^3/mol)
S	1	3380	14.1	12
N	1	1000	4.18	-9
6 member ring	1	250	1.05	16
Phenylene	2	7630	31.9	52.4
$-CH_2-$	2	1180	4.94	16.1
Total		22250	93.1	156

Table S6. Fedors group contribution of the phthalimide pendant on P3

Group	Quantity	E (cal/mol)	E (kJ/mol)	V_m (cm^3/mol)
Phenylene	1	7630	31.9	52.4
C=O	2	4150	17.4	10.8
N	1	1000	4.18	-9
5 member ring	1	250	1.05	16
$-CH_2-$	2	1180	4.94	16.1
Total		19540	81.8	113.2

Table S7. Fedors group contribution of the phthalimide pendant on P3 in the charged state.

Group	Quantity	E (cal/mol)	E (kJ/mol)	V_m (cm^3/mol)
Phenylene	1	7630	31.9	52.4
CON	1	7050	29.5	-7.7
C-O	1	1150	4.81	-15.4
5 member ring	1	250	1.05	16
$-CH_2-$	2	1180	4.94	16.1
Total		18440	77.2	77.5

Table S8. Calculated δ and χ of the polymers.

Polymer	P1	P1 Charged	P2	P2 Charged	P3	P3 Charged
δ ($MPa^{1/2}$)	22.3	20.4	22.7	22.7	23.4	24.4
χ	0.527	0.909	0.468	0.468	0.396	0.343

Table S9. Calculated δ and χ of the polymer ion pairs

Polymer ion pair	P1	P2	P3
δ ($MPa^{1/2}$)	19.9	21.7	20.6
χ	1.055	0.614	0.873

Table S10. Calculated δ and χ of the P2 polymer and ion pair in DCM.

Polymer	P2	P2 ion pair
χ	0.486	0.394

Table S11. Calculated electrochemical properties and Flory-Huggins solvent interaction parameters of P2 in DCM.

$D_{app, CA}$ ionization (cm^2 s^{-1})	$D_{app, CA}$ neutralization (cm^2 s^{-1})	$D_{phys, DLS}$ (cm^2 s^{-1})	$k_{ex, ionization}$ (cm^{-1} s^{-1})	$k_{ex, neutralization}$ (cm^{-1} s^{-1})	χ neutral	χ ionized
1.1×10^{-6}	7.6×10^{-7}	3.8×10^{-7}	1.8×10^{12}	9.6×10^{11}	0.486	0.486

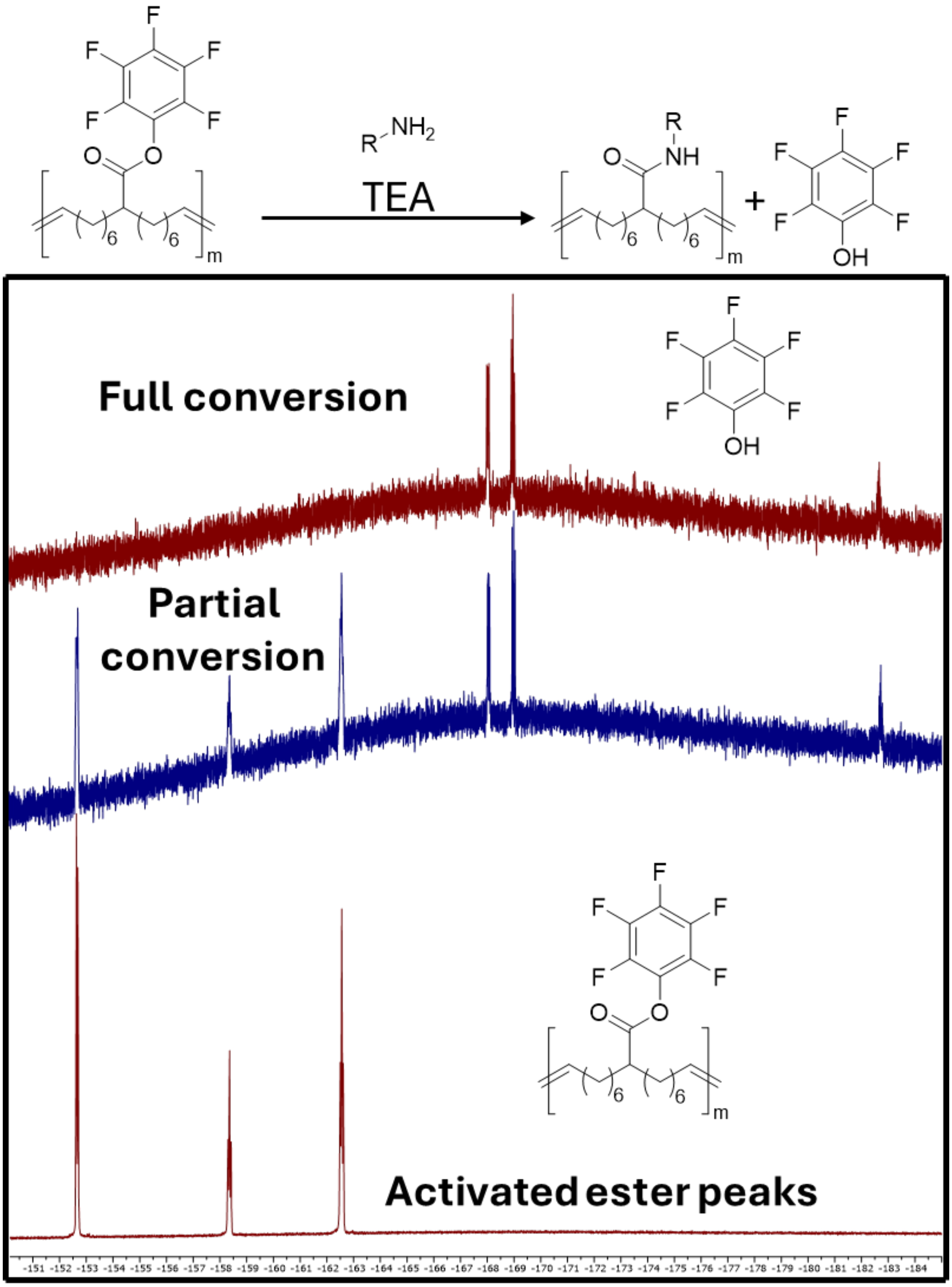

Figure S1. ^{19}F NMR showing conversion from activated ester pendant by observing in situ bound pentafluorophenyl peaks disappearing and free pentafluorophenol peaks appearing over time.

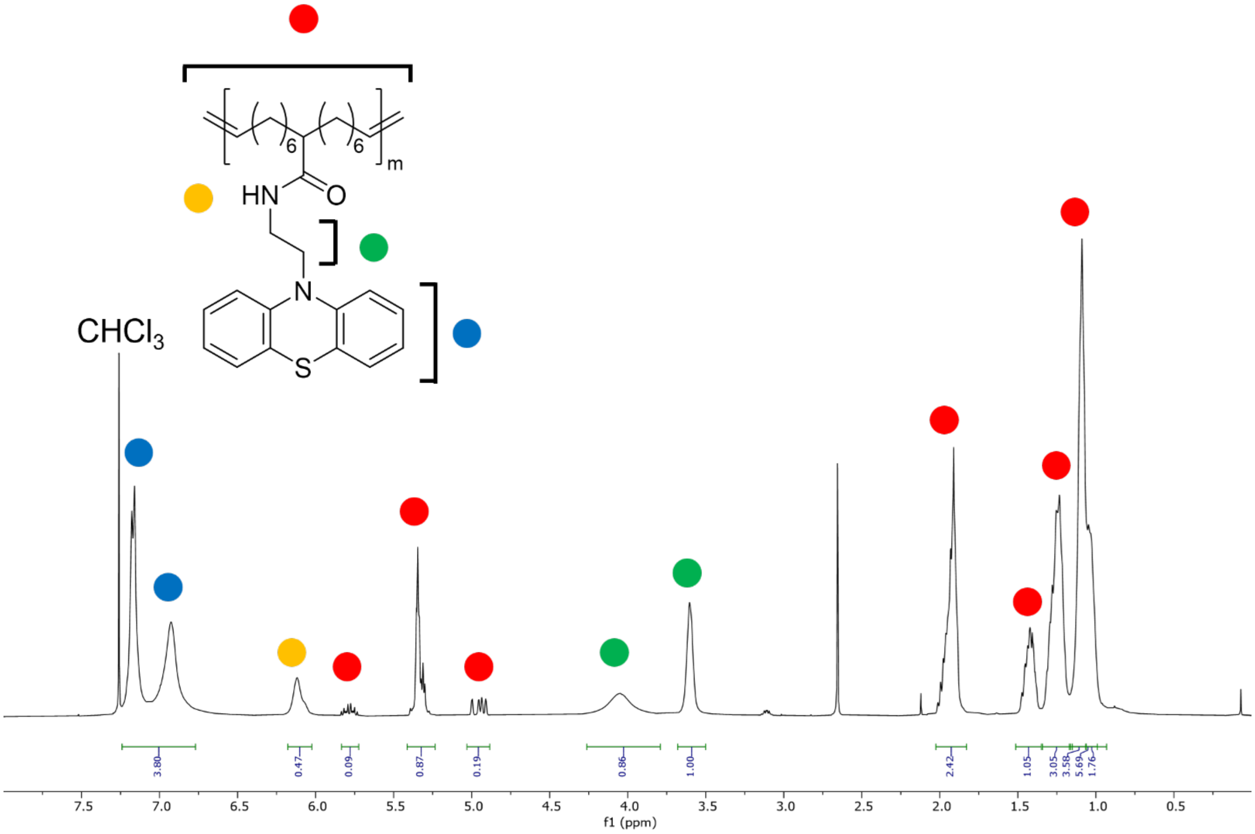

Figure S2. ^{1}H NMR of P2 in $CDCl_3$.

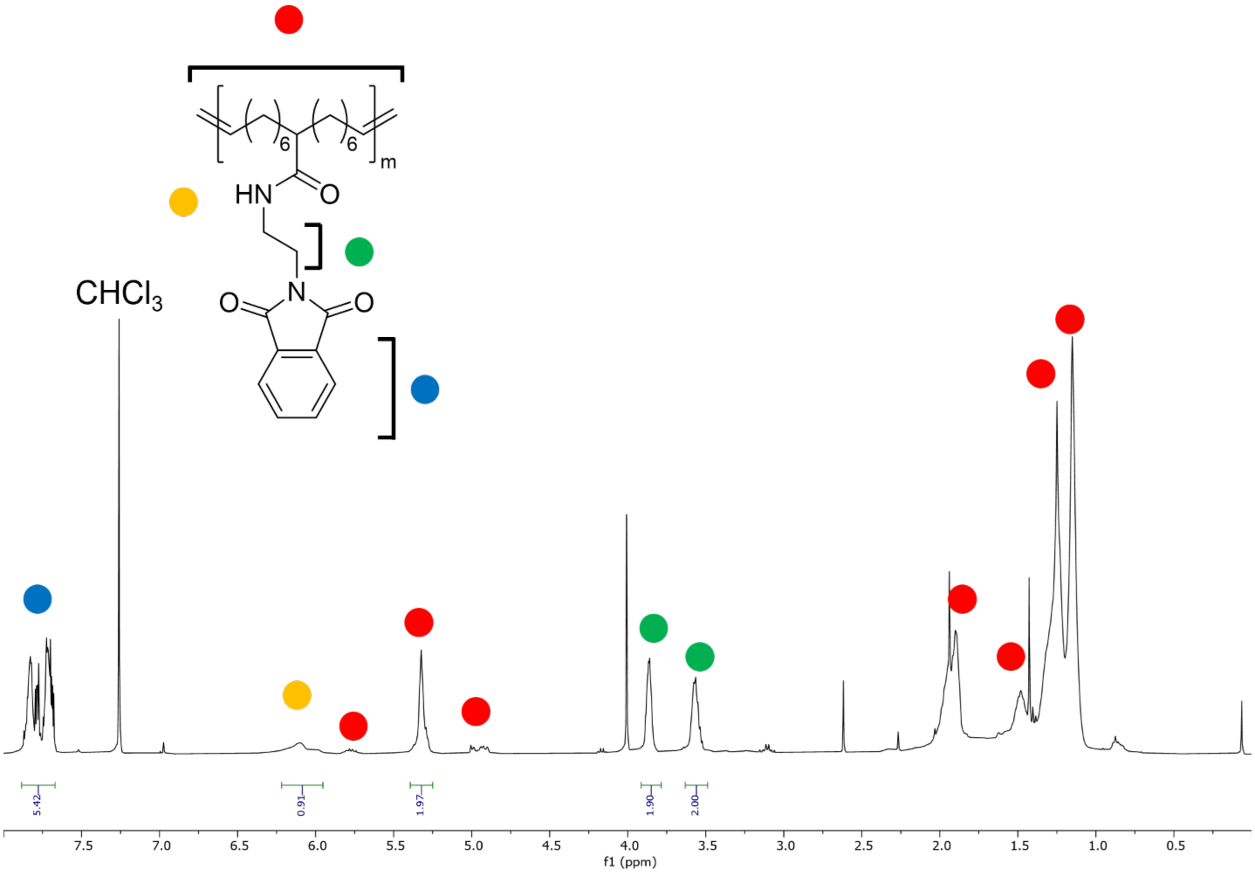

Figure S3. ^{1}H NMR of P3 in $CDCl_3$.

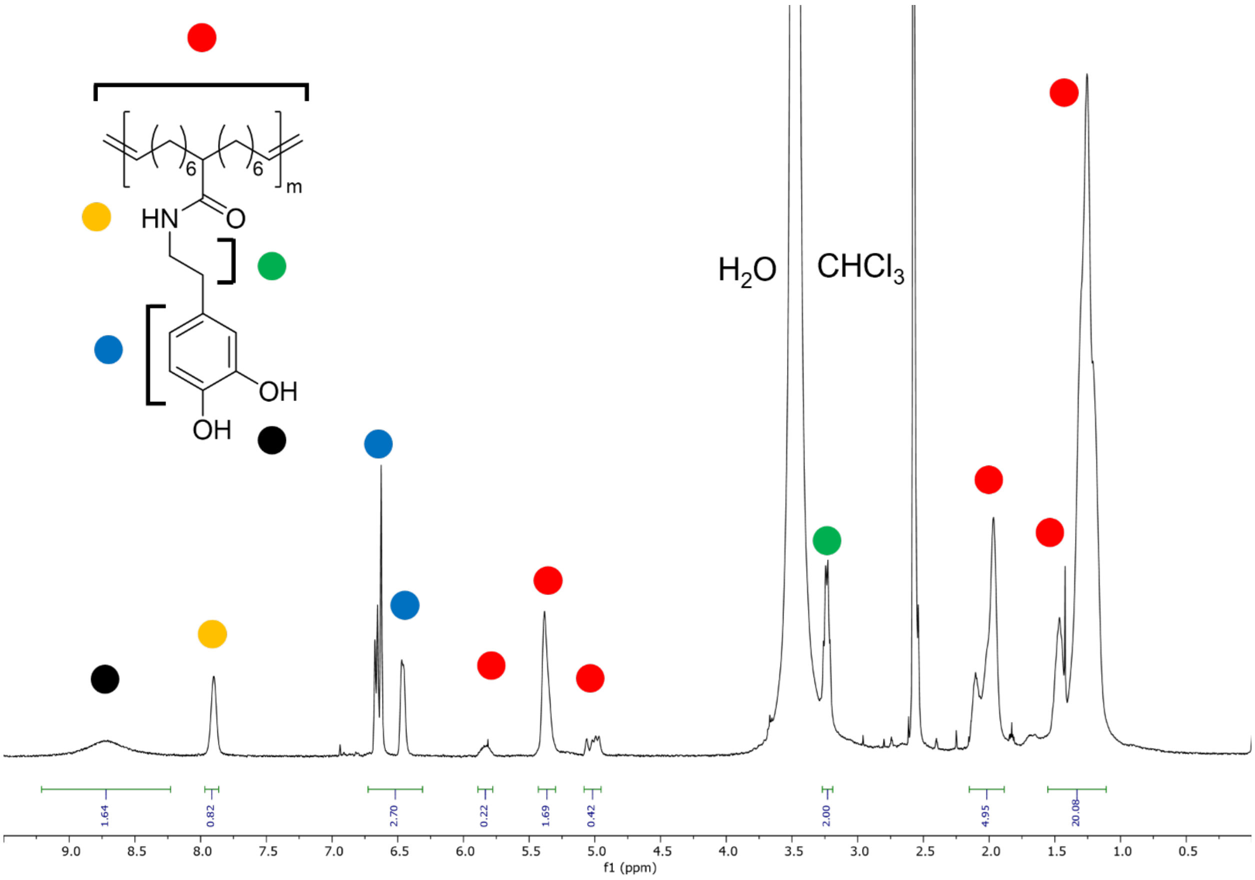

Figure S4. ^1H NMR of P4 in DMSO-d6. Sample was difficult to obtain due to poor solubility. Signals were only able to be observed in sufficient magnitude after increasing intensity using analyzing software.

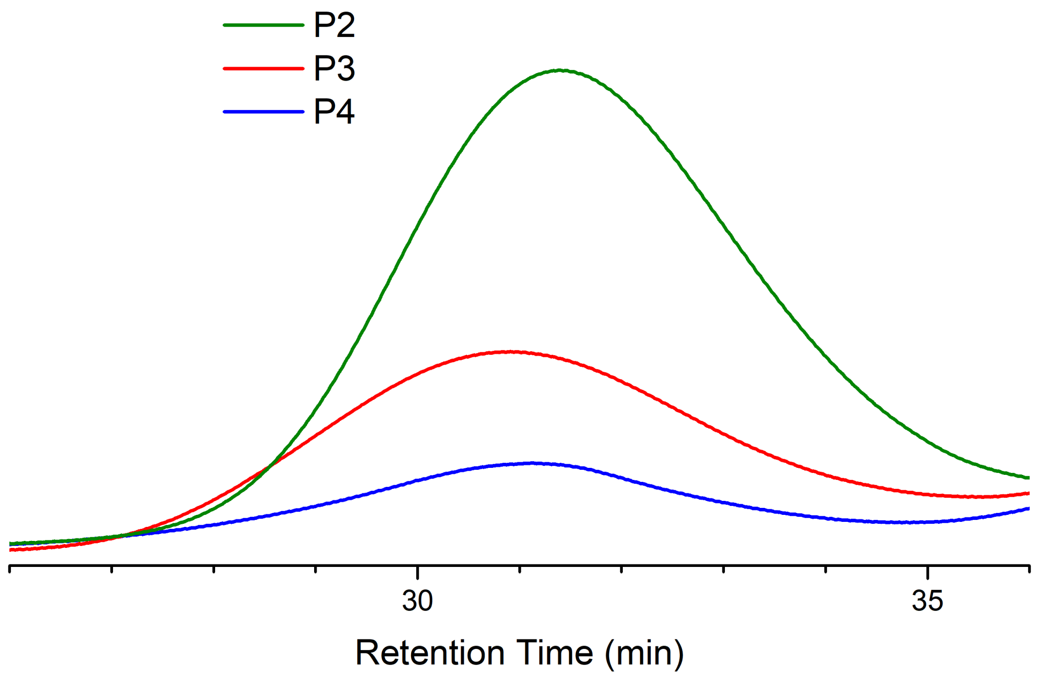

Figure S5. GPC/SEC traces of polymers prepared from n = 6 backbone (P-AE).

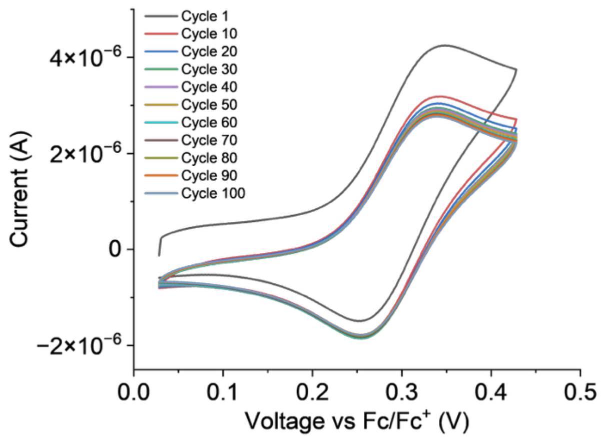

Figure S6. Cyclic voltammogram of P2 (2 mM) in 0.5 M TBAPF6 in DMF at 20 mV/s for 100 cycles in a three-electrode beaker cell configuration (WE: GCE, RE: Ag/AgNO3, and CE: Pt wire).

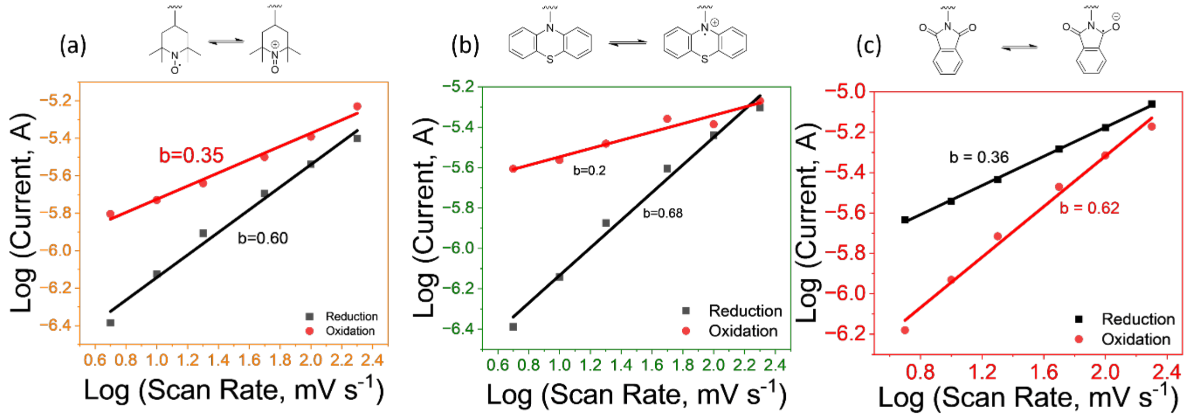

Figure S7. Log-log plots of peak current and scan rates of three polymers. The slope of the line (b-value) is shown. Tests are done using a 2 mM solution of the polymers (by repeat unit) in an electrolyte consists of 0.5M $TBAPF_6$ in DMF. The b-values smaller than 0.5 indicate adsorption of polymers onto the glassy carbon surface.[8]

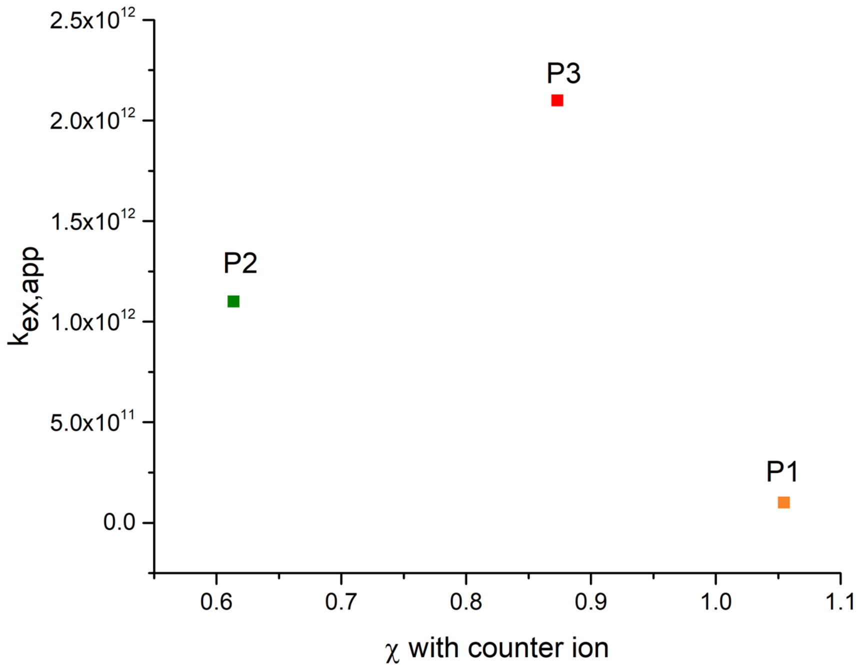

Figure S8. Graph of $k_{ex,app}$ in the neutralization step vs χ of the prepared polymers with their respective counter ion.

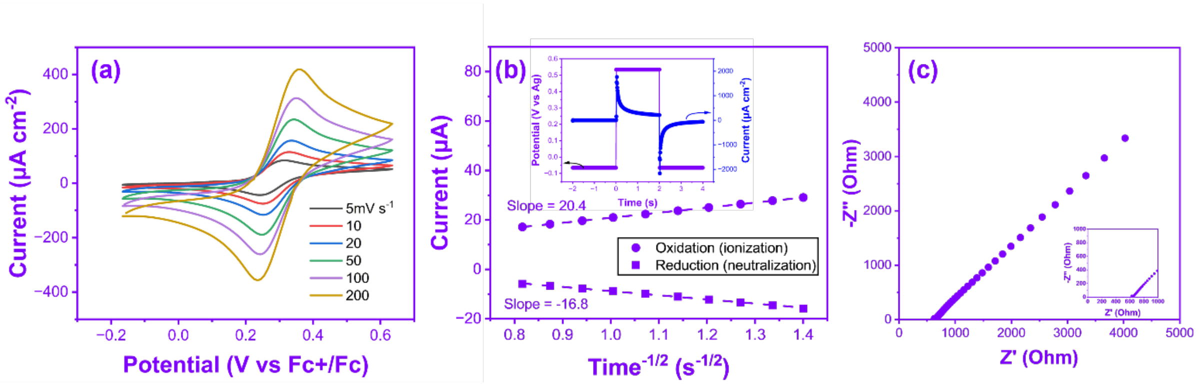

Figure S9. a) Cyclic voltammograms referenced to ferrocene, b) Cottrell plot, and c) EIS Nyquist plot results of P2 conducted in DCM. CV setup: 2 mM solution (by repeating unit) in 0.5 M $TBAPF_6$ in DCM, 3 mm diameter glassy carbon working electrode, platinum wire counter electrode, and silver wire reference electrode. Working potentials are converted using a ferrocene internal reference. EIS test was performed at $E_{1/2}$ for polymer using a frequency range of 0.1 Hz to 100 kHz and an amplitude of 5mV. Inset shows the high frequency region of the Nyquist plots.

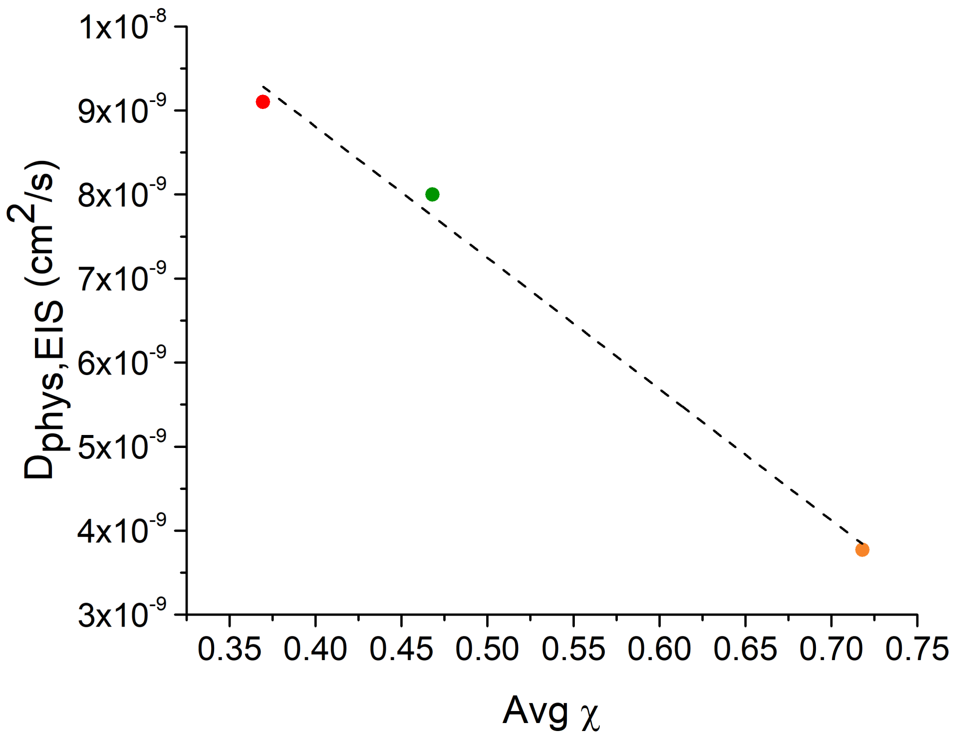

Figure S10. $D_{phys,EIS}$ plotted against the average χ of the neutral and charged states of the prepared polymers. Because EIS was done at $E_{1/2}$ of each polymer, the redox active sites are assumed to be half ionized/half neutral, here we used the average χ of the ionized and neutral polymers to resemble the polymer-solution affinity under this condition. Line to guide reader's eye.

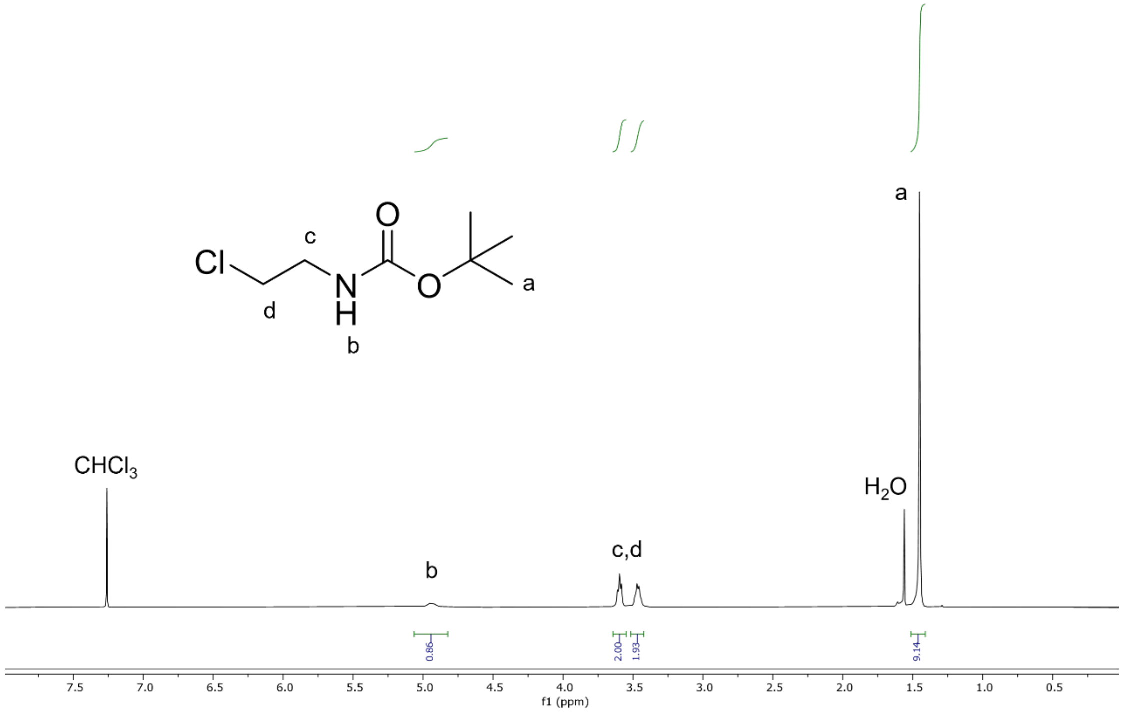

Figure S11. ^{1}H NMR of tertbutyl (2-chloroethyl) carbamate in $CDCl_3$.

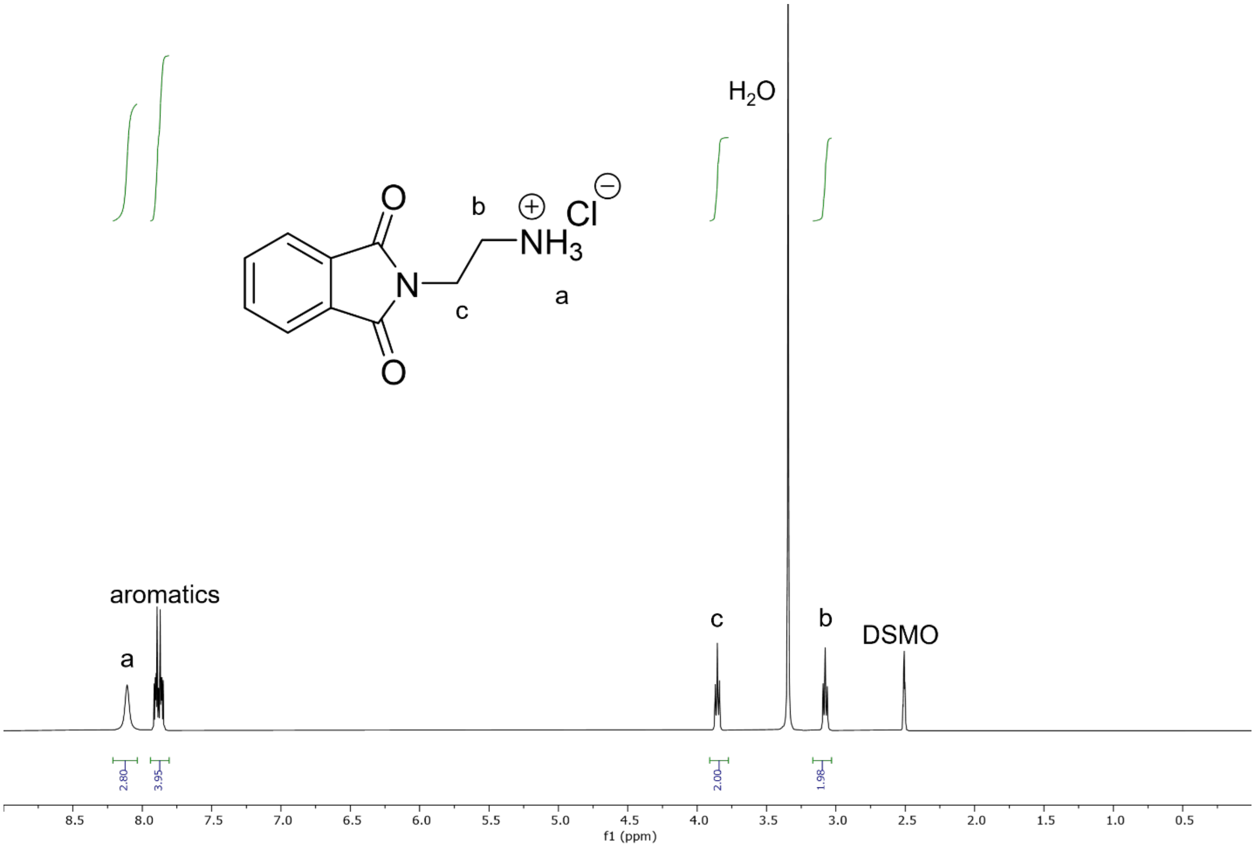

Figure S12. ^{1}H NMR of 2-(2-aminoethyl)isoindoline-1,3-dione HCl in DMSO-d6.

References

(1) Chankeshwara, S. V.; Chakraborti, A. K. Catalyst-Free Chemoselective N-Tert-Butyloxycarbonylation of Amines in Water. *Org. Lett.* **2006**, *8* (15), 3259–3262. https://doi.org/10.1021/ol0611191.

(2) Johnson, D.; Hussain, J.; Bhoir, S.; Chandrasekaran, V.; Sahrawat, P.; Hans, T.; Khalil, M. I.; Benedetti, A. D.; Thiruvenkatam, V.; Kirubakaran, S. Synthesis, Kinetics and Cellular Studies of New Phenothiazine Analogs as Potent Human-TLK Inhibitors. *Org. Biomol. Chem.* **2023**, *21* (9), 1980–1991. https://doi.org/10.1039/D2OB02191A.

(3) Ma, T.; Fox, E.; Qi, M.; Li, C.-H.; Sachithani, K. A. N.; Mohanty, K.; Tabor, D. P.; Pentzer, E. B.; Lutkenhaus, J. L. Charge Transfer in Spatially Defined Organic Radical Polymers. *Chem. Mater.* **2023**. https://doi.org/10.1021/acs.chemmater.3c02148.

(4) Grignon, E.; An, S. Y.; Battaglia, A. M.; Seferos, D. S. Catechol Homopolymers and Networks through Postpolymerization Modification. *Macromolecules* **2022**, *55* (22), 10167–10175. https://doi.org/10.1021/acs.macromol.2c01513.

(5) Fedors, R. F. A Method for Estimating Both the Solubility Parameters and Molar Volumes of Liquids. *Polym. Eng. Sci.* **1974**, *14* (2), 147–154. https://doi.org/10.1002/pen.760140211.

(6) Easley, A. D.; Vukin, L. M.; Flouda, P.; Howard, D. L.; Pena, J. L.; Lutkenhaus, J. L. Nitroxide Radical Polymer–Solvent Interactions and Solubility Parameter Determination. *Macromolecules* **2020**, *53* (18), 7997–8008. https://doi.org/10.1021/acs.macromol.0c01739.

(7) Barton, A. F. M. *CRC Handbook of Solubility Parameters and Other Cohesion Parameters*, 2nd ed.; Routledge, 2017. https://doi.org/10.1201/9781315140575.

(8) Albagli, D.; Bazan, G.; Wrighton, M. S.; Schrock, R. R. Well-Defined Redox-Active Polymers and Block Copolymers Prepared by Living Ring-Opening Metathesis Polymerization. *J. Am. Chem. Soc.* **1992**, *114* (11), 4150–4158. https://doi.org/10.1021/ja00037a017.

(9) Trachioti, M. G.; Lazanas, A. Ch.; Prodromidis, M. I. Shedding Light on the Calculation of Electrode Electroactive Area and Heterogeneous Electron Transfer Rate Constants at Graphite Screen-Printed Electrodes. *Microchim. Acta* **2023**, *190* (7), 251. https://doi.org/10.1007/s00604-023-05832-w.
